# A study for multiscale information transfer measures based on conditional mutual information

**DOI:** 10.1371/journal.pone.0208423

**Published:** 2018-12-06

**Authors:** Xiaogeng Wan, Lanxi Xu

**Affiliations:** Department of Mathematics, College of Science, Beijing University of Chemical Technology, Beijing, China; University of British Columbia, CANADA

## Abstract

As the big data science develops, efficient methods are demanded for various data analysis. Granger causality provides the prime model for quantifying causal interactions. However, this theoretic model does not meet the requirement for real-world data analysis, because real-world time series are diverse whose models are usually unknown. Therefore, model-free measures such as information transfer measures are strongly desired. Here, we propose the multi-scale extension of conditional mutual information measures using MORLET wavelet, which are named the WM and WPM. The proposed measures are computational efficient and interpret information transfer by multi-scales. We use both synthetic data and real-world examples to demonstrate the efficiency of the new methods. The results of the new methods are robust and reliable. Via the simulation studies, we found the new methods outperform the wavelet extension of transfer entropy (WTE) in both computational efficiency and accuracy. The features and properties of the proposed measures are also discussed.

## 1 Introduction

As big data science developments, practical time series methods are demanded to study the complexity and dynamics of the data. Real-world data are time series usually obtained by experiments or observations whose models are diverse and the data are often nonlinear or non-stationary [1–2], e.g. the EEG time series measured from experiments [2–5] and financial data observed from real-world markets [6]. Therefore, efficient method is necessary to study the dynamics of these complex systems.

Various directed methods have been developed for studying the directed interaction between time series. The most classic causality measure is the Granger causality (GC) [7–8], it is a prime model for causality measures which uses significance tests to detect the directed dependency of one time series on another time series [7–8]. However, GC has many limitations, it is bivariate time domain method suits for only linear models [7–8]. Other similar methods have been derived to cover the limitation of GC [9–12]. For instance, the conditional Granger causality is a multivariate method that can detect direct interactions between time series [10], the frequency domain GC is derived for frequency domain data analysis [10–11], and the nonlinear GC can be applied to nonlinear data analysis [12]. More advanced measures are such as the Partial Direct Coherence (PDC) [13–17] and Directed Transfer Function (DTF) [18–19], which are sophisticated frequency domain measures. However, both PDC and DTF are linear measures whose validity rely on the linear autoregressive or moving average model fitting [13–19].

Since real world time series are often nonlinear and sometimes nonstationary, the data models are usually varied and unknown, therefore linear causal measures are farfetched to analyze the real-world time series. To analyze the dynamics and interactions between real-world time series and also theoretic models, information transfer measures are preferred [1, 12, 20–30]. Information transfer measures are used to detect the directed information transfer between coupled time series which can be used to study the direction of interactions of complex networks. However, the information transfer is a different notion to the causal effect [31–34]. In [33], J. T. Lizier and M. Prokopenko have used the study of the transfer entropy [27] and information flow to differentiate the concepts of information transfer and causal effect [33]. N. Ay and D. Polani have introduced in [31] the notion of causal independence which allows for defining a measure for the strength of a causal effect. In their work, they call this notion the information flow which is compared with the transfer entropy. A relevant work is presented by Wibral et. al. in [34], in which an extension method from transfer entropy is proposed that account for delayed source-target interactions, while crucially retaining the conditioning on the embedded target state at the immediately previous time step [34]. This new extension method proposed by Wibral et. al. in [34] is proved as the only relevant option in keeping with the Wiener's principle of causality. To clarify the notional causality which is different from notion of information transfer, a clear and systematic literature of causality, including theories and causal models is presented in the work by J. Pearl in [32].

Transfer entropy (TE) is a fundamental information transfer measure proposed by T. Schreiber [27], it is a directed information transfer method that evaluates the bivariate information transfer between coupled time series. Due to the model-free nature of information methods, information transfer measures such as transfer entropy (TE) are preferred in many studies to analyze the interactions for varied models. To suffice the needs of different analysis, many other information transfer methods are derived from the transfer entropy. For instance, the symbolic transfer entropy (STE) [28] and the Partialized Transfer Entropy (PTE) [35] are derived to improve TE for particular applications. However, these transfer entropy measures are computationally redundant, in that they use uniform embeddings in their expressions, which leads to high computational complexity and redundancy in their computations, because variables of no significant contribution to the information transfer detection are also included in the computation [22, 29].

To solve this problem, non-uniform state-space embedding methods such as MIME (conditional mutual information from mixed embeddings) [29] and partial MIME (PMIME, a direct version of MIME) [22] are developed to reduce the computational redundancies. Both MIME and PMIME use a progressive scheme of a maximum criterion and a stopping criterion to select significant contributive components from uniform state-space embedding vector to form refined embedding vectors for information transfer detection [22, 29]. In consequence, both methods are computational efficient and have wide applications to various data analysis [22, 26, 29, 35–38].

Most of these measures require data stationarity [7–8, 13–19]. Real world data such as financial and biological time series are not seriously stationary. Wavelet is an ideal tool for non-stationary data analysis who presents good solutions to time and frequency allocations and outperforms the short-time Fourier transforms [24, 39–43]. In [24], MORLET wavelet [40] is introduced to TE to cover non-stationary and discontinuous data analysis [24]. Since TE is primitive and computationally redundant, we are inspired to use MORLET wavelet to extend MIME and PMIME into computational efficient multi-scale measures that cover the deficiency of WTE, and the new extensions are expected to be useful in various data analysis particular the real-world data analysis.

In this paper, we introduce the MORLET wavelet extension to MIME and PMIME and study their efficiency in application to both model data and real-world time series. The paper is organized as follows. In the Introduction section, we review the background of this study. In the section of Materials and methods, we describe the formulative wavelet extension of MIME and PMIME. In the Results section, four synthetic models (theoretic maps) and two real-world examples (EEG and financial data) are used to demonstrate the efficiency of the proposed extensions, where all simulation studies are compared to the wavelet-extension of TE (WTE). In the Discussion section, application and features of the new methods are discussed. The final conclusion of this study is drawn in the Conclusion section.

## 2 Materials and methods

In this section, we introduce the details of the wavelet extension of MIME and PMIME. Here, we refer the two wavelet extensions as WM and WPM, respectively.

### 2.1 MORLET wavelet extension of MIME (WM)

Conditional mutual information from mixed embeddings (MIME) [29] is a time domain nonlinear information transfer measure whose wavelet extension is described as follows.

Assume X and Y are two arbitrary time series of length N, *L*_*X*_ and *L*_*Y*_ denote the maximum time lag for X and Y. To compute the WM for Y->X, a mother function $\psi (\eta )=\pi^{-1/4}e^{i\omega_{0}\eta }e^{-\eta^{2}/2}$ [2, 24, 39–43] is used to convert the X and Y time series into MORLET wavelet coefficients [24]

$$V_{s_{i},\tau_{X}}=\frac{1}{\sqrt{s_{i}}}\sum_{t=1}^{N}X_{t}\psi^{*}(\frac{t-\tau_{X}}{s_{i}})$$ (1)

$$W_{s_{i},\tau_{Y}}=\frac{1}{\sqrt{s_{i}}}\sum_{t=1}^{N}Y_{t}\psi^{*}(\frac{t-\tau_{Y}}{s_{i}})$$ (2)

where *ω*_0_ ∈ [5,6] is the normalized frequency, time lags *τ*_*X*_ (1 ≤ *τ*_*X*_ ≤ *L*_*X*_) and *τ*_*Y*_ (1 ≤ *τ*_*Y*_ ≤ *L*_*Y*_) are the translation parameters used to localize the wavelet, *s*_*i*_ (1 ≤ i ≤ m, m is the total number of time scales) is the time scale that determines the width and resolution of the wavelet, * represents the complex conjugation [24]. This wavelet setting is the same to the WTE [24].

The WM are computed for every time scale *s*_*i*_ (1 ≤ i ≤ m). For each time scale *s*_*i*_ (1 ≤ i ≤ m), a future embedding vector of X of time horizon T [29] ($T\in N^{+}$) is defined as

$$V_{F}(s_{i})=(V_{s_{i},\tau +1},V_{s_{i},\tau +2},\cdots ,V_{s_{i},\tau +T});$$ (3)

A collective set of candidate components is also defined at the time scale:

$$B(s_{i})=(V_{s_{i},\tau },V_{s_{i},\tau -1},\cdots ,V_{s_{i},\tau -L_{X}},W_{s_{i},\tau },W_{s_{i},\tau -1},\cdots ,W_{s_{i},\tau -Y});$$ (4)

The same progressive scheme of MIME [29] is used for each time scale *s*_*i*_. The progressive scheme starts with an empty vector **b**_0_(*s*_*i*_) = ∅. In the first iterative cycle, WM goes through B(*s*_*i*_) to find the element *x*′ that satisfies the maximum criterion [29]:

$$I:\;I(x^{\prime };V_{F}(s_{i}))=\max_{x\in B(s_{i})}I(x;V_{F}(s_{i})),\;x^{\prime }\in B(s_{i}).$$ (5)

where *I*(*x*;*V*_*F*_(*s*_*i*_)) is the mutual information rate between the x and *V*_*F*_(*s*_*i*_). The element *x*′ that satisfies the maximum criterion is selected to join **b**_0_(*s*_*i*_) that forms ***b***_1_(*s*_*i*_) = (*x*′), *x*′ is then removed from B(*s*_*i*_) and obtains B_*k*−1_(*s*_*i*_) = B(*s*_*i*_)\{*x*′} [29].

At a k-th iterative cycle, WM seeks the element *x*′ in the remaining set B_*k*−1_(*s*_*i*_) (obtained from the k-1-th iterative cycle) that satisfies the maximum criterion [29]

$$I:\;I(x^{\prime };V_{F}(s_{i})|b_{k-1}(s_{i}))=\max_{x\in B_{k-1}(s_{i})}I(x;V_{F}(s_{i})|b_{k-1}(s_{i})),\;x^{\prime }\in B_{k-1}(s_{i}),$$ (6)

and moves the element *x*′ from B_*k*−1_(*s*_*i*_) to ***b***_*k*−1_(*s*_*i*_) to obtain the enlarged embedding vector ***b***_*k*_(*s*_*i*_) = (*x*′,***b***_*k*−1_(*s*_*i*_)) and B_*k*_(*s*_*i*_) = B_*k*−1_(*s*_*i*_)\{*x*′}.

The progressive scheme stops at a k+1-th iterative cycle and uses *b*_*k*_(*s*_*i*_) as the final selected embedding vector, if the following stopping criterion is satisfied [29]:

$$\frac{I(b_{k}(s_{i});V_{F}(s_{i}))}{I(b_{k+1}(s_{i});V_{F}(s_{i}))}>A,$$ (7)

Here, A is the significance threshold (between 0 and 1) controls the inclusion of embedding components [29]. This stopping criterion ensures contributive components to be included while prevents useless components from being added. The progressive scheme stops if no significant information can be given when including new component is included [29, 35–38].

The WM for time scale *s*_*i*_ is evaluated by [29]:

$${WM}_{Y\to X}(s_{i})=1-\frac{I(V_{F}(s_{i});b_{k}^{V}(s_{i}))}{I(V_{F}(s_{i});b_{k}(s_{i}))}=\frac{I(V_{F}(s_{i});b_{k}^{W}(s_{i})|b_{k}^{V}(s_{i}))}{I(V_{F}(s_{i});b_{k}(s_{i}))}$$ (8)

We note that this WM information transfer between coupled time series is evaluated at the same time scale *s*_*i*_, (i = 1,2,…,64), which means that the WM does not evaluate the cross scales information transfers. This is limitation of this method. The evaluation of cross-scale information transfers will be our research of the next stage.

### 2.2 MORLET wavelet extension PMIME (WPM)

WPM is the multi-variate version of WM, which inferences only the direct interactions. Without the loss of generality, assume X, Y and Z are three arbitrary time series of length N, *L*_*X*_, *L*_*Y*_, *L*_*Z*_ are the maximum time lags for the three time series. WPM first converts the X,Y, Z time series into MORLET wavelet coefficients [24]:

$$V_{s_{i},\tau_{X}}=\frac{1}{\sqrt{s_{i}}}\sum_{t=1}^{N}X_{t}\psi^{*}(\frac{t-\tau_{X}}{s_{i}})$$ (9)

$$W_{s_{i},\tau_{Y}}=\frac{1}{\sqrt{s_{i}}}\sum_{t=1}^{N}Y_{t}\psi^{*}(\frac{t-\tau_{Y}}{s_{i}})$$ (10)

$$U_{s_{i},\tau_{Z}}=\frac{1}{\sqrt{s_{i}}}\sum_{t=1}^{N}Z_{t}\psi^{*}(\frac{t-\tau_{Z}}{s_{i}})$$ (11)

where the mother function and all the other wavelet parameters are the same to WM.

WPM values are computed for every time scale. To compute the WPM of Y->X, a future embedding vector [22] of time horizon T is defined:

$$V_{F}(s_{i})=(V_{s_{i},\tau +1},V_{s_{i},\tau +2},\cdots ,V_{s_{i},\tau +T}).$$ (12)

Different from WM, the collective set of candidate components are multivariate that are defined by all time series in the system [22]:

$$B(s_{i})=(V_{s_{i},\tau },V_{s_{i},\tau -1},\cdots ,V_{s_{i},\tau -L_{X}},W_{s_{i},\tau },W_{s_{i},\tau -1},\cdots ,W_{s_{i},\tau -Y},U_{s_{i},\tau },U_{s_{i},\tau -1},\cdots ,U_{s_{i},\tau -Z});$$ (13)

The initial selected embedding vector is again an empty vector **b**_0_(*s*_*i*_) = ∅.

WPM follows the same progressive scheme and the maximum criterion to WM with the only difference of the collective set of candidate components. WPM selects candidate components from all rather than two variables in the system which contribute to the inference of direct information transfers [22].

If the progressive scheme stops at a k+1-th iterative cycle and uses **b**_*k*_(*s*_*i*_) as the final selected embedding vector. The WPM for Y->X at time scale *s*_*i*_ is given by

$${WPM}_{Y\to X}(s_{i})=\frac{I(V_{F}(s_{i});b_{k}^{W}(s_{i})|b_{k}^{V}(s_{i}),b_{k}^{U}(s_{i}))}{I(V_{F}(s_{i});b_{k}(s_{i}))}$$ (14)

where $b_{k}^{V}(s_{i}),\;b_{k}^{W}(s_{i})$ and $b_{k}^{U}(s_{i})$ are the X, Y and Z components of **b**_*k*_(*s*_*i*_) [22, 29], respectively. Also, we should note that the WPM evaluates information transfer between wavelet coefficients of the same scales.

### 2.3 Bias correction by surrogate data

We use time-shifted surrogate [22,24,29,44–49,50] to test the significance of the results. Take WM as an example. Let $\left\{V_{s_{i},\tau_{X}}\right\}$ and $\left\{W_{s_{i},\tau_{Y}}\right\}$ to denote the MORLET wavelet coefficients for the two arbitrary time series X and Y, the *WM*_*X*→*Y*_(*s*_*i*_) indicates the WM information transfer from X to Y evaluated at time scale *s*_*i*_. We fix $\left\{V_{s_{i},\tau_{X}}\right\}$ and permute the temporal indices of $\left\{W_{s_{i},\tau_{Y}}\right\}$ randomly [22,24,29,50] to obtain the surrogate of $\left\{W_{s_{i},\tau_{Y}}\right\}$. Next, we apply the WM method on the original series of $\left\{V_{s_{i},\tau_{X}}\right\}$ and the surrogate time series of $\left\{W_{s_{i},\tau_{Y}}\right\}$, the results are denoted as *WM*_*X*→*Y*_(*s*_*i*_,*q*), where q is the index for the surrogates of $\left\{W_{s_{i},\tau_{Y}}\right\}$. Thus, the bias corrected WM for *X*→*Y* is given by [24]

$${WM}_{C,X\to Y}(s_{i})={WM}_{X\to Y}(s_{i})-\max_{q}{\{WM}_{X\to Y}(s_{i},q)\}$$ (15)

In the following context, we use *WM*_*C*,*X*→*Y*_(*s*_*i*_) to denote the bias corrected WM information transfer from X to Y [22,24,29,50]. The bias corrected WM for the inverse direction, and the bias corrected WPM and WTE are similarly defined. In all simulations, we use q = 10 [22,24,29,50].

### 2.4 Contrast information transfer

To obtain the dominance of interaction between coupled time series, we compute the contrast information transfer between paired variables [24]. For example, to analyze the dominance of interaction between two time series X and Y, we compute the contrast information transfer between *X*→*Y* and *Y*→*X*for each time scale *s*_*i*_: Ω_*WM*,*X*→*Y*_(*s*_*i*_) = *WM*_*C*,*X*→*Y*_(*s*_*i*_)−*WM*_*C*,*Y*→*X*_(*s*_*i*_). If Ω_*WM*,*X*→*Y*_(*s*_*i*_)>0, the dominant information transfer is detected for *X*→*Y*; and vice versa, if Ω_*WM*,*Y*→*X*_(*s*_*i*_)>0, the dominant information transfer is detected for *Y*→*X*. The contrast information transfer for WPM and WTE are similarly defined.

## 3 Results

In this section, we use six examples, including both synthetic data and real-world time series, to demonstrate the efficiency of WM and WPM. In these examples, various types of interactions are displayed, and all simulation studies are compared with the method of WTE.

### 3.1 Synthetic data

Synthetic data are examples of model time series generated by equations. These examples include the Henon maps, linear autoregressive models, and Lorenz systems [22, 29, 50–53], which are indeterministic systems that are frequently used for time series analysis [22, 29, 50, 53]. Here, we use four synthetic examples to demonstrate the efficiency of the proposed information transfer measures.

#### 3.1.1 Cosine map

The cosine map consists of two unidirectionally coupled first order autoregressive processes where one of the processes contains a cosine [24]:

$$\begin{array}{c} x_{t+1}=0.7x_{t}+0.7\;\cos\left(0.3t\right)+n_{t}^{\left(x\right)}\left(0,\sigma^{2}\right),\\ y_{t+1}=0.7y_{t}+n_{t}^{\left(y\right)}\left(0,\sigma^{2}\right)+ex_{t}, \end{array}$$ (16)

where $n_{t}^{\left(x\right)},n_{t}^{\left(y\right)}$ are independent zero mean Gaussian random processes with variance *σ*^2^ = 2, and e∈[0,1] is the coupling strength controls the linear interaction from *X*_*t*_ to *Y*_*t*_. The data of this example can be found in S1 Dataset.

The initial data are randomly generated from normal distribution with zero mean and unit variance. Each data series contains 5×10^4^ data points. For comparison purpose, we use the same MORLET parameters for WM and WPM as recommended for WTE [24, 40]: r = 0.125,*ω*_0_ = 6,*s*_0_ = 0.5,*V* = 10, and *n* = 64.

To analyze the effect of stopping criteria, we compute the WM and WPM as functions of the stopping criterion A. The stopping criterion A represents the proportionality of the conditional mutual information between the current and the previous iterative cycles. In general applications [22,29,46], A is a value close to but no greater than 1 [22, 29]. Larger values of A representing looser criteria, while smaller values of A imply more rigid criteria. In the MIME and PMIME studies, the usual choice of the stopping criteria is A = 0.95 for MIME and A = 0.97 for PMIME. These choices of A value are obtained by various simulation studies, A = 0.95 and 0.97 are appropriate A values that not only allow useful lagged values to be detected but also prevent false positiveness from being included [22,29,46]. To study the impact of the stopping criteria A on evaluation of the information transfer, we alter the criterion A = 0.91, 0.93, 0.95, 0.97, 0.99, and present the WM and WPM results (directional inference) in Table 1. Since the cosine map has only two processes, the multivariate measure WPM has same results to that of the bivariate measure WM. We can see from Table 1, when the coupling strength 0.1≤e≤1, both WM and WPM indicate the correct information transfer from X to Y. When e = 0, the coupling disappears and no flow of information is detected.

**Table 1:** WM information transfer for the cosine map with different stopping criteria and coupling strengths.

**Coupling strength**	**A = 0.91**	**A = 0.93**	**A = 0.95**	**A = 0.97**	**A = 0.99**
**e = 0**	-	-	-	-	-
**0.1≤e≤1**	X->Y	X->Y	X->Y	X->Y	X->Y

This table shows the directional inference of the cosine map by using WM at different stopping criteria A and coupling strength e. Since the cosine map is bivariate, the WM and WPM have the same results on this example.

**Fig 1:**
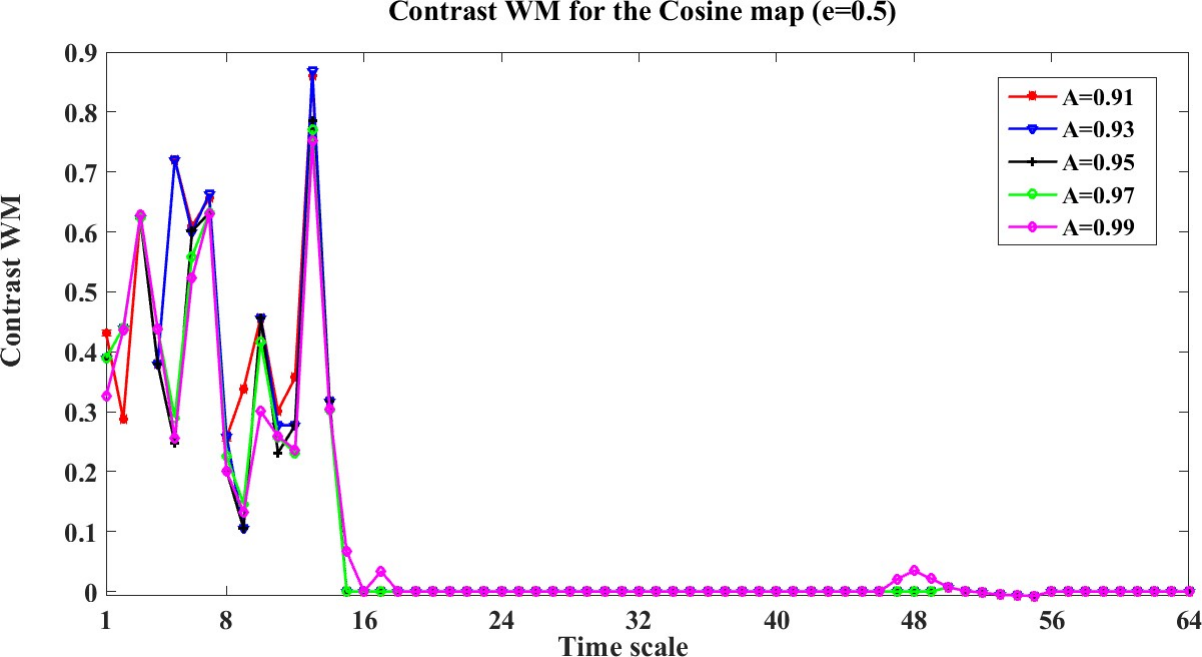
The contrast WM for the Cosine map at different stopping criteria A (e = 0.5). This figure shows the contrast WM Ω_*WM*,*X*→*Y*_(*s*_*i*_,*e*) = *WM*_*C*,*X*→*Y*_(*s*_*i*_,*e*)−*WM*_*C*,*Y*→*X*_(*s*_*i*_,*e*)(i = 1,2,…64,e = 0,0.1,0.2,…,1) for the cosine map (coupling e = 0.5). The curves with different colors represent the contrast WM at different stopping criteria (A = 0.91, 0.93,0.95,0.97,0.99).

An example of the contrast WM results between X and Y (coupling strength e = 0.5) at different scales are shown in Fig 1. In this figure, we can see that different stopping criteria A presents similar results of the contrast WM between X and Y. However, the stopping criterion theoretically becomes looser when A increases. In later Henon map analysis, we will see that A = 0.95 and A = 0.97 are good choices, but A = 0.95 is a bit rigid than A = 0.97 in the directional inference of indirect interactions, therefore we use A = 0.97 for both WM and WPM in all simulation studies.

**Fig 2:**
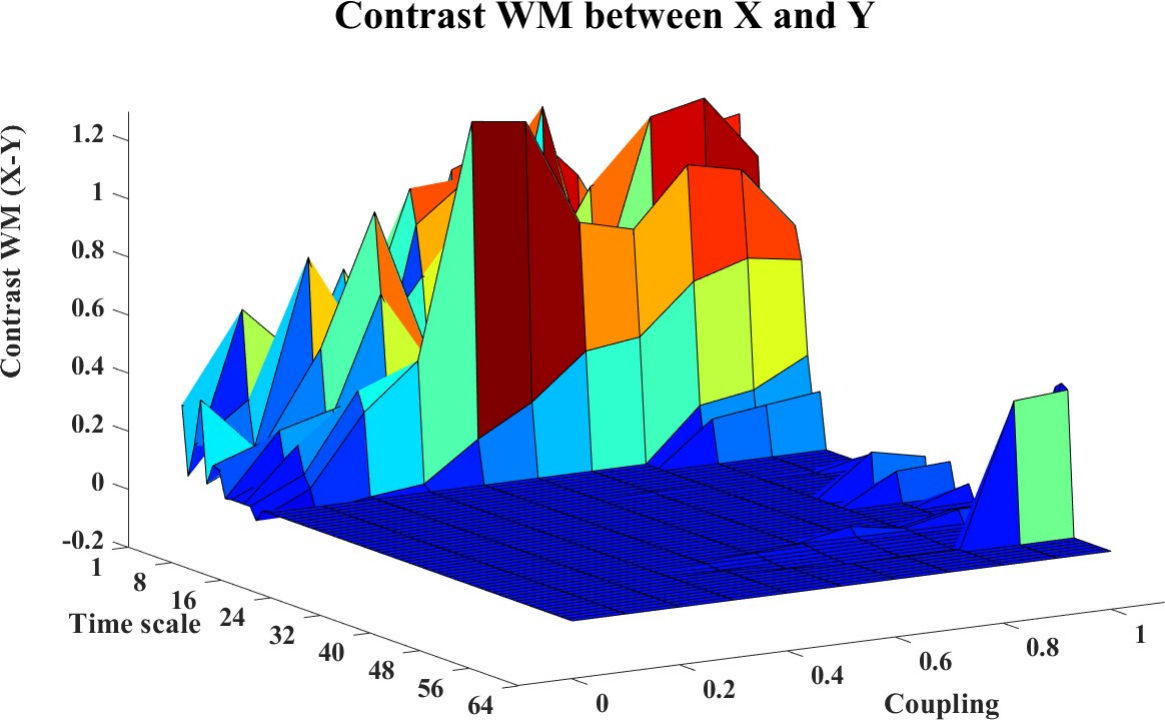
The contrast WM information transfer values for the cosine map. This figure shows the 3D surface of the contrast WM Ω_*WM*,*X*→*Y*_(*s*_*i*_,*e*) = *WM*_*C*,*X*→*Y*_(*s*_*i*_,*e*)−*WM*_*C*,*Y*→*X*_(*s*_*i*_,*e*)(i = 1,2,…64,e = 0,0.1,0.2,…,1) for the cosine map. This surface represents the values of the contrast information transfer against different time scale *s*_*i*_ and coupling strength e. The non-negative surface implicates the directed influence from X->Y.

**Fig 3:**
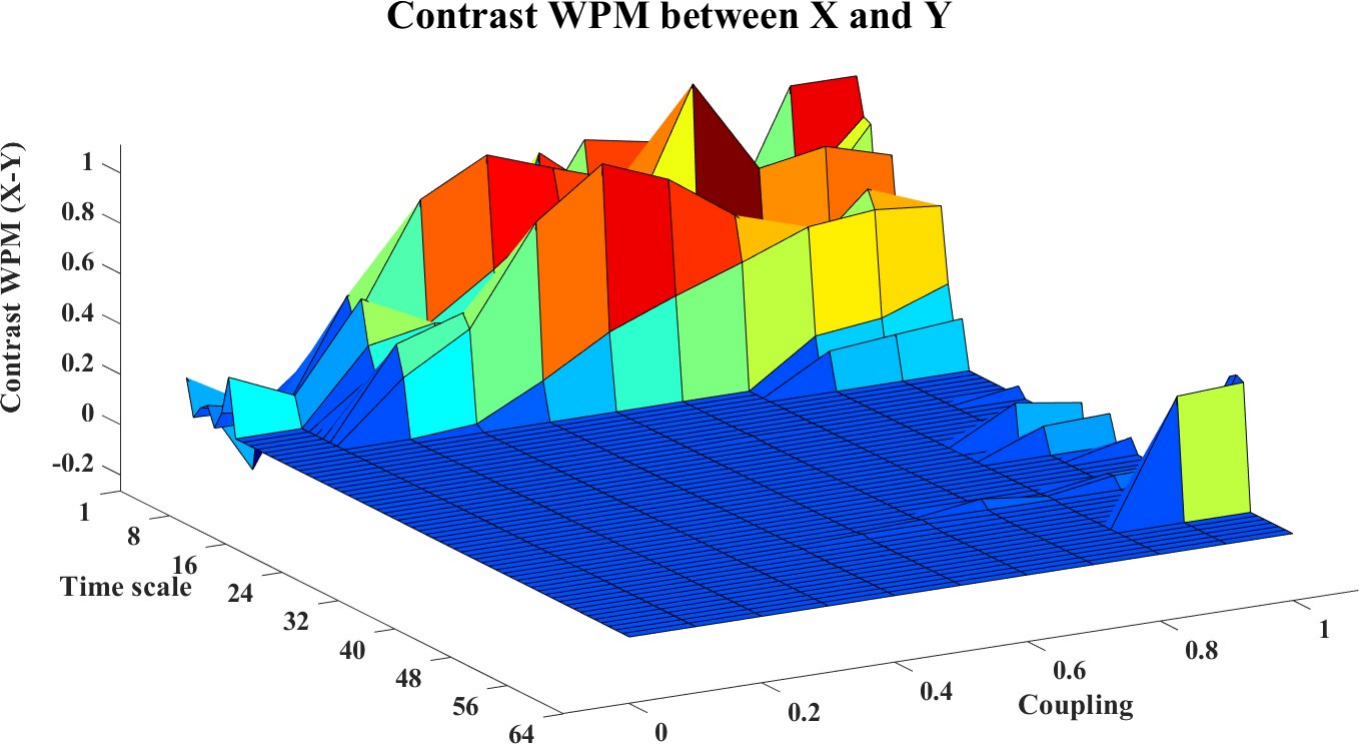
The contrast WPM information transfer values for the cosine map. This figure shows the 3D surface of the contrast WPM Ω_*WPM*,*X*→*Y*_(*s*_*i*_,*e*) = *WPM*_*C*,*X*→*Y*_(*s*_*i*_,*e*)−*WPM*_*C*,*Y*→*X*_(*s*_*i*_,*e*)(i = 1,2,…64,e = 0,0.1,0.2,…,1) for the cosine map. The surface represents the values of the corrected information transfer against time scale *s*_*i*_ and coupling strength e. The non-negative surface implies the directed information transfer from X->Y.

**Fig 4:**
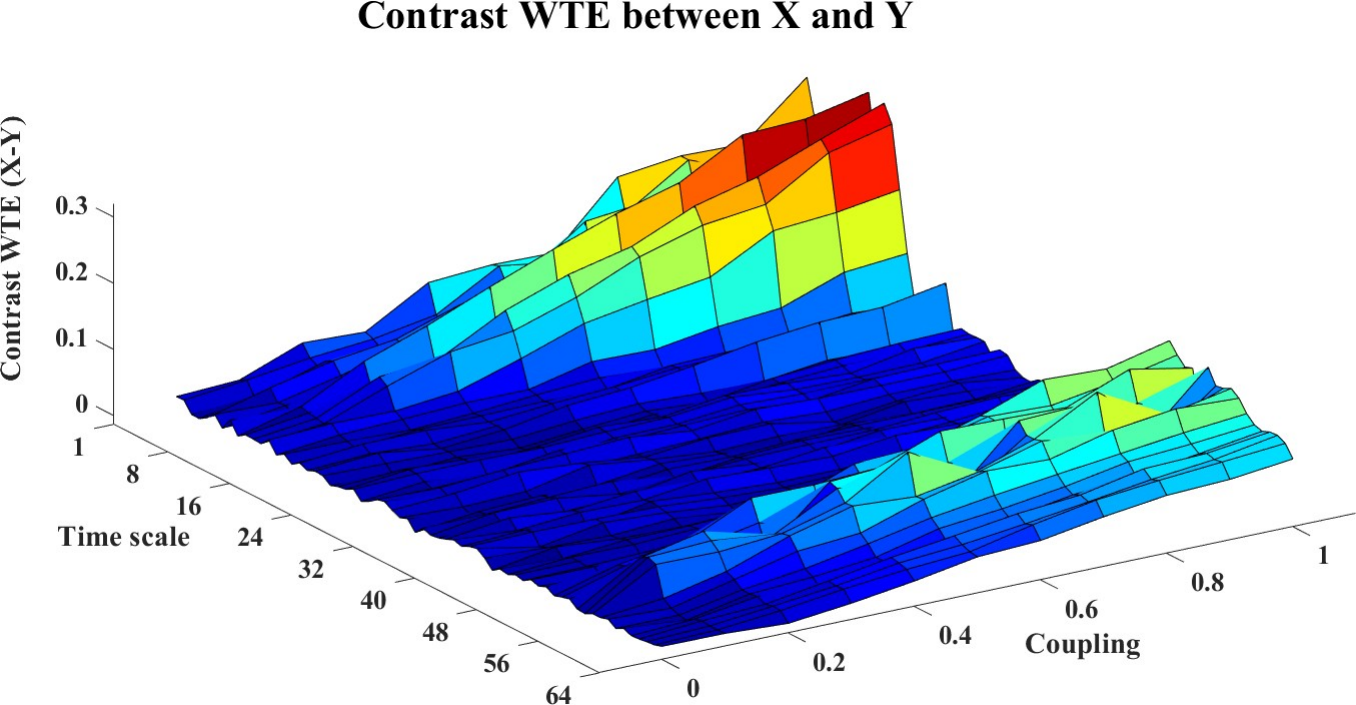
The contrast WTE information transfer values for the cosine map. In this figure, the 3D surface presents the contrast WTE Ω_*WTE*,*X*→*Y*,_(*s*_*i*_,*e*) = *WTE*_*C*,*X*→*Y*_(*s*_*i*_,*e*)−*WTE*_*C*,*Y*→*X*_(*s*_*i*_,*e*)(i = 1,2,…64,e = 0,0.1,0.2,…,1) for the cosine map at different time scale *s*_*i*_ and coupling strength e. The non-negative surface indicates the directed information transfer from X->Y.

In this example, we use the stopping criterion A = 0.97 and the referenced embedding parameters [22, 29, 36–38]: T = 1 (time horizon, prediction time step) and Lmax = 5 (the maximum time lag). The WM, WPM and WTE information transfer values are filtered with the surrogate bias correction. Moreover, the contrast information transfer values between X and Y are computed for WM, WPM and WTE which are shown in Figs 2–4, respectively.

In these figures, the contrast information transfer values are plotted against time scales and coupling strength. We can see that both all three measures inference the correct linear interaction from X→Y, which are supported by the non-negative surfaces of Ω_*WM*,*X*→*Y*_ (Fig 2), Ω_*WPM*,*X*→*Y*_ (Fig 3), and Ω_*WTE*,*X*→*Y*_ (Fig 4).

In these figures, we can see a ‘cosine’ shape (a slowly increasing ridge or a ‘tail’ rather than well-localized in scale) of the surfaces, which nicely reflects the ‘cosine’ influence on the dynamics of the coupled system [24].

**Fig 5:**
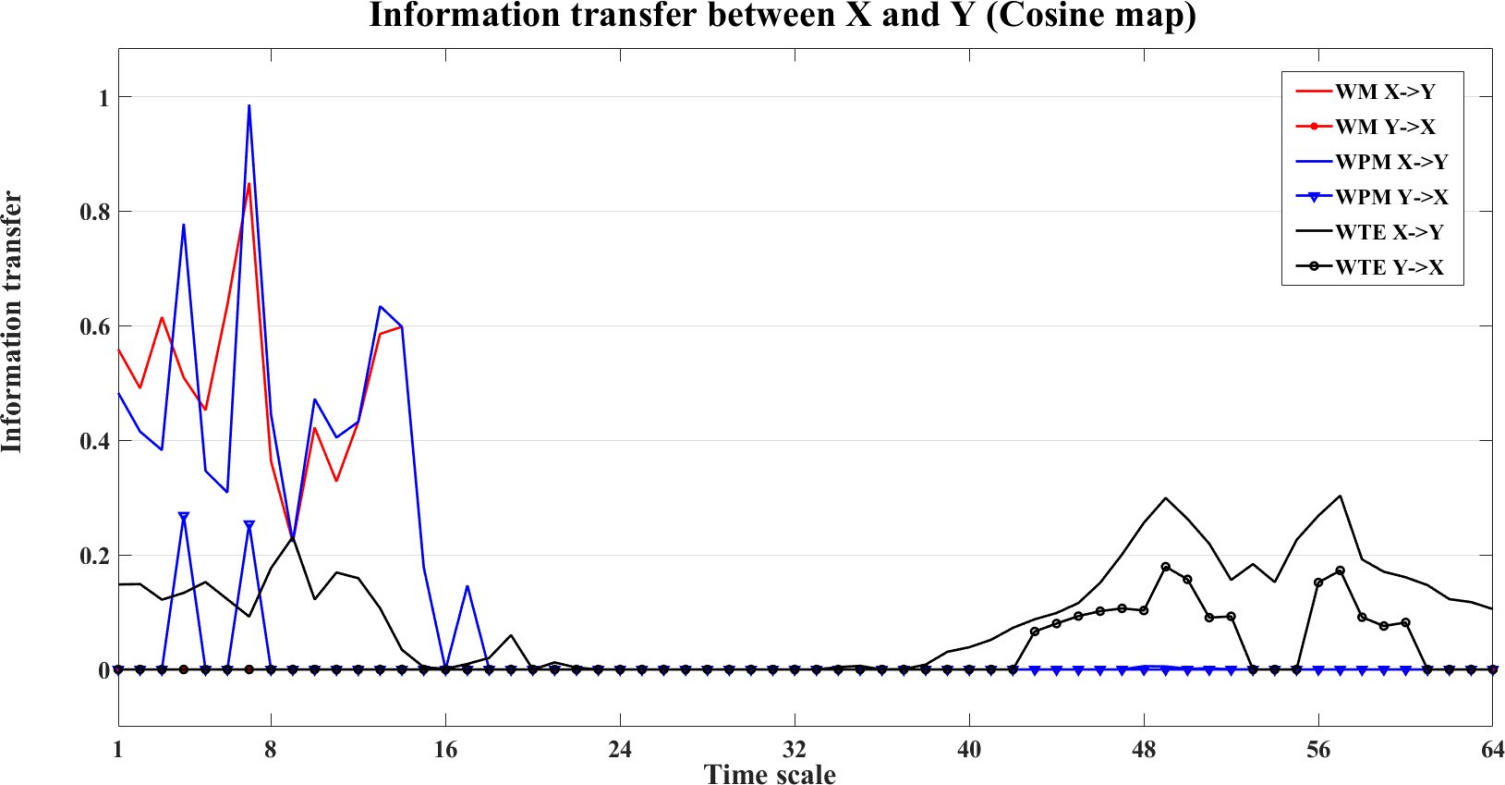
Information transfer between X and Y. In this figure, we present the WM (red), WPM (blue) and WTE (black) information transfer values of cosine map (coupling strength e = 0.8) in each independent direction. We can see that all measures present dominant information transfer values for X->Y.

To see the information transfer in each independent direction, we plot the bias corrected information transfer values in Fig 5.

#### 3.1.2 Four unidirectionally coupled Henon maps

The four unidirectionally coupled Henon maps are defined by the equations [22,24–25,29,36–38,51–52]:

$$\begin{array}{l} X_{1,n+1}=1.4-x_{1,n}^{2}+0.3X_{1,n-1},\\ X_{2,n+1}=1.4-0.3X_{1,n}X_{2,n}-0.7X_{2,n}^{2}+0.3X_{2,n-1},\\ X_{3,n+1}=1.4-0.3X_{2,n}X_{3,n}-0.7X_{3,n}^{2}+0.3X_{3,n-1},\\ X_{4,n+1}=1.4-0.3X_{3,n}X_{4,n}-0.7X_{4,n}^{2}+0.3X_{4,n-1}, \end{array}$$ (17)

the direct nonlinear directed interactions are from *X*_*i*−1_→*X*_*i*_,*i* = 2,3,4. The wavelet parameters used are r = 0.125,*ω*_0_ = 6,*s*_0_ = 0.2,*V* = 8, and *n* = 64 [24]. The other WM and WPM parameters are the same to the previous example. The data of this example can be found in S2 Dataset.

These Henon maps have more than two variables, the WPM results are different from that of the WM. To study the influence of the stopping criteria on the multivariate example, we make the following analysis on the direct interaction between X2 and X3, and also the indirect interaction between X1 and X3. Results of the directional inference of different stopping criteria A is shown in Table 2. In this table, both WM and WPM detect all correct interactions between the unidirectionally coupled Henon maps. When the stopping criteria is small (A≤0.95), WM detects only direct interactions, when A≥0.97, WM also detects the indirect interactions from X1->X3, X2->X4. WPM is a direct measure, so it detects only the direct interactions for all the different stopping criteria.

**Table 2:** Directional inference of WM and WPM with different stopping criteria.

Stopping criteria	WM	WPM
**A = 0.91**	X1->X2, X2->X3, X3->X4	X1->X2, X2->X3, X3->X4
**A = 0.93**	X1->X2, X2->X3, X3->X4	X1->X2, X2->X3, X3->X4
**A = 0.95**	X1->X2, X2->X3, X3->X4	X1->X2, X2->X3, X3->X4
**A = 0.97**	X1->X2, X2->X3, X3->X4,X1->X3, X2->X4	X1->X2, X2->X3, X3->X4
**A = 0.99**	X1->X2, X2->X3, X3->X4,X1->X3, X2->X4	X1->X2, X2->X3, X3->X4

This table shows the directional inference of WM and WPM between the four unidirectionally coupled Henon maps with different stopping criteria A. We can see that WPM detects only the direct interactions, while WM also detects indirect direction when A≥0.97.

**Fig 6:**
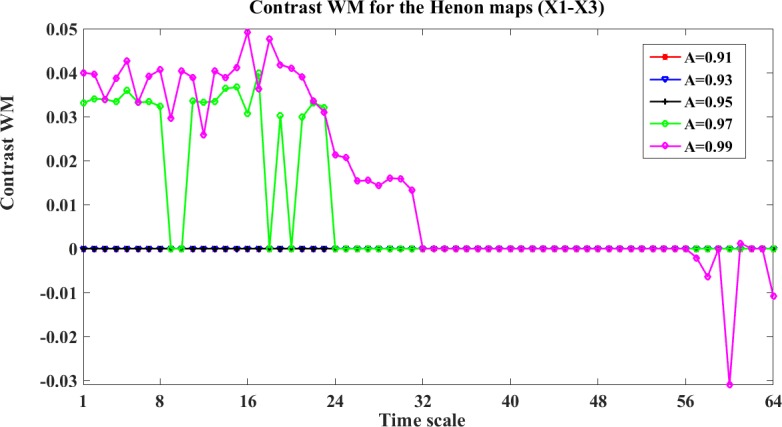
The contrast WM for the four unidirectionally coupled Henon maps with different stopping criteria (X1-X3). This figure shows the line plots of the contrast WM (contrast values between X1 and X3) Ω_*WM*,*X*1→*X*3_(*s*_*i*_,*e*) = *WM*_*C*,*X*1→*X*3_(*s*_*i*_,*e*)−*WM*_*C*,*X*3→*X*1_(*s*_*i*_,*e*)(i = 1,2,…64) for the Henon maps between X2 and X3. This curves with different colors represent the contrast WM results obtained by different stopping criteria.

To analyze the influence of the stopping criteria, we take X1 and X3 as an example. Fig 6 shows the contrast WM between X1 and X3 with different stopping criteria and time scales. In this figure, the indirect interaction from X1->X3 can only be detected when A≥0.97, and when A = 0.99, WM presents false positiveness for X3->X1. Because WPM is a direct measure, it gives all zeros for the information transfer between X1 and X3 for all stopping criteria.

**Fig 7:**
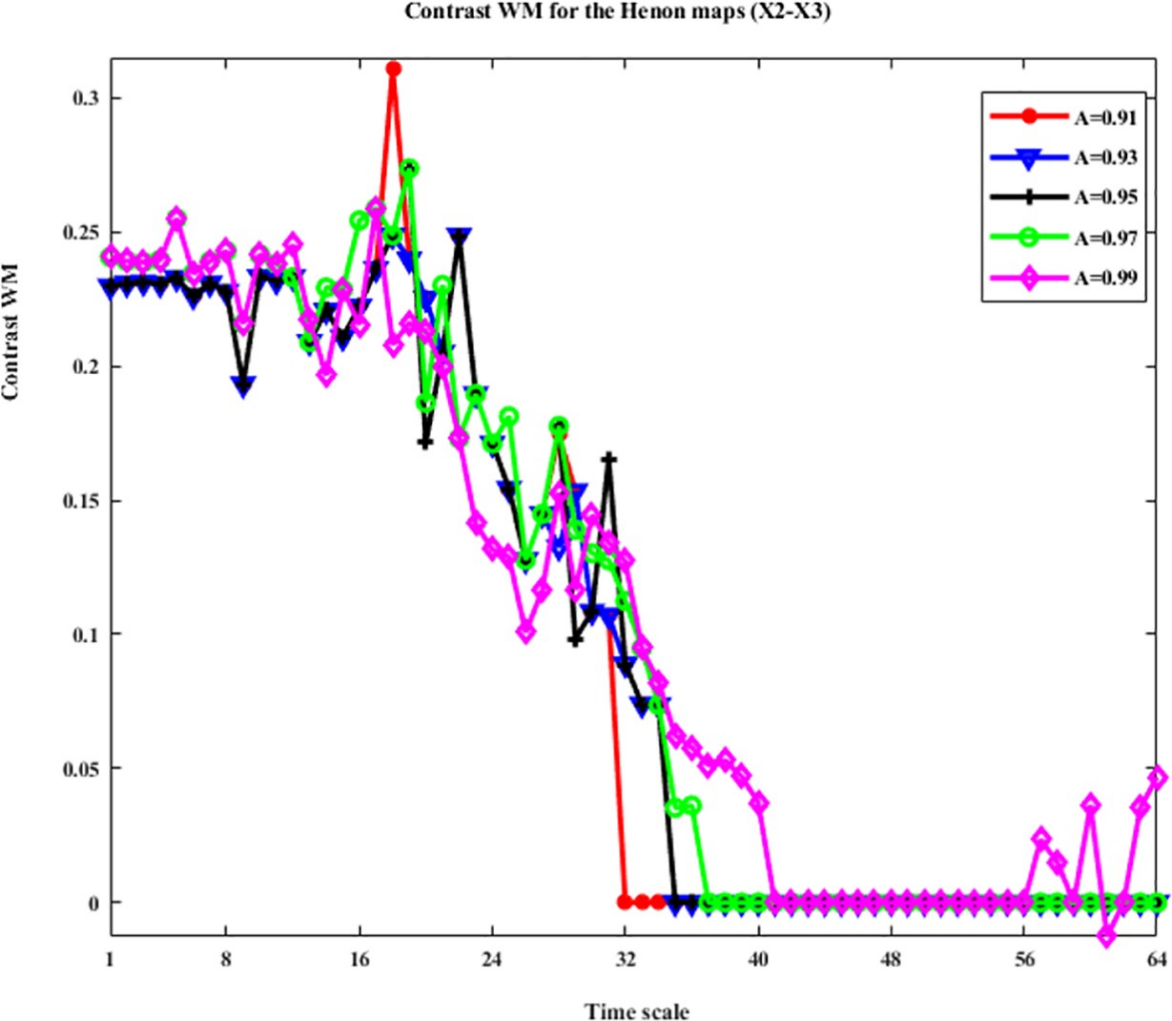
The contrast WM for the four unidirectionally coupled Henon maps with different stopping criteria (X1-X3). This figure shows the contrast WM between X1 and X3: Ω_*WM*,*X*1→*X*3_(*s*_*i*_,*e*) = *WM*_*C*,*X*1→*X*3_(*s*_*i*_,*e*)−*WM*_*C*,*X*3→*X*1_(*s*_*i*_,*e*)(i = 1,2,…64) with different stopping criteria and time scales. This curves with different colors represent the contrast WM obtained by different stopping criteria.

**Fig 8:**
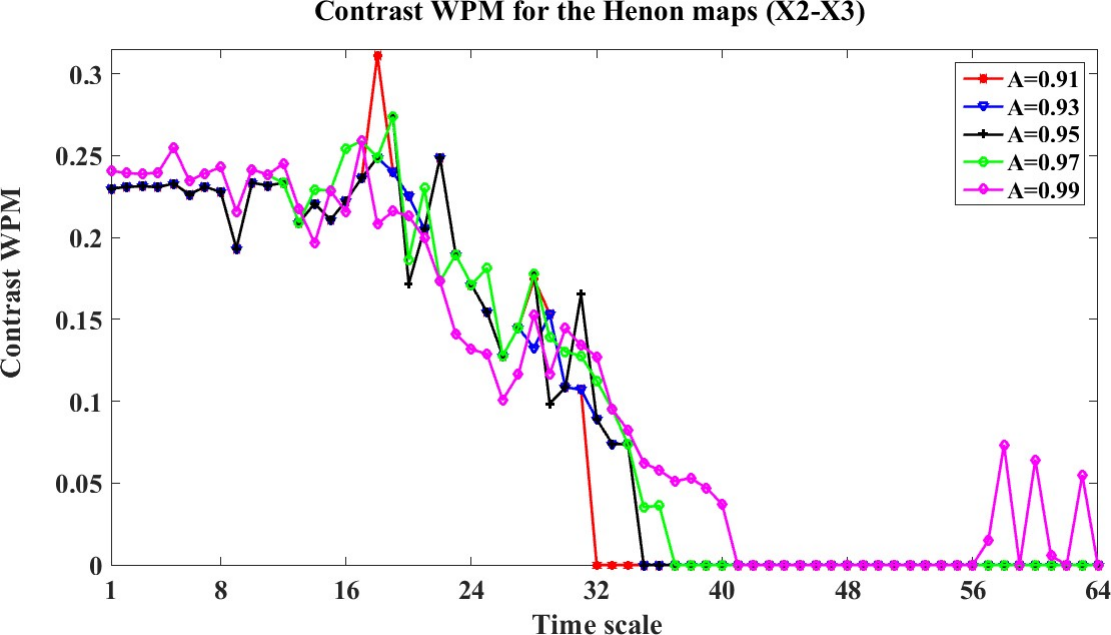
The contrast WPM for the four unidirectionally coupled Henon maps with different stopping criteria (X1-X3). This figure shows the contrast WM between X1 and X3: Ω_*WPM*,*X*1→*X*3_(*s*_*i*_,*e*) = *WPM*_*C*,*X*1→*X*3_(*s*_*i*_,*e*)−*WPM*_*C*,*X*3→*X*1_(*s*_*i*_,*e*)(i = 1,2,…64) with different stopping criteria and time scales. This curves with different colors represent the contrast WPM obtained by different stopping criteria.

Similarly, we analyze the criteria effect on the direct interactions. We take the direct interaction X2->X3 as an example. The WM and WPM information transfer between X2 and X3 with different stopping criteria A are shown in Figs 7 and 8. We can see from these figures, the different stopping criteria presents similar values of the contrast results for both WM and WPM, and the trends of the contrast WM and the contrast WPM are similar. This is because this interaction from X2->X3 is direct, and the WM and WPM may present similar results. Also, we note that in the contrast results of WM, when A = 0.99 the contrast WM presents negative values which implies a false direction from X3->X2. This maybe because the A is large and the stopping criteria become too loose that false positive is detected in this situation.

**Fig 9:**
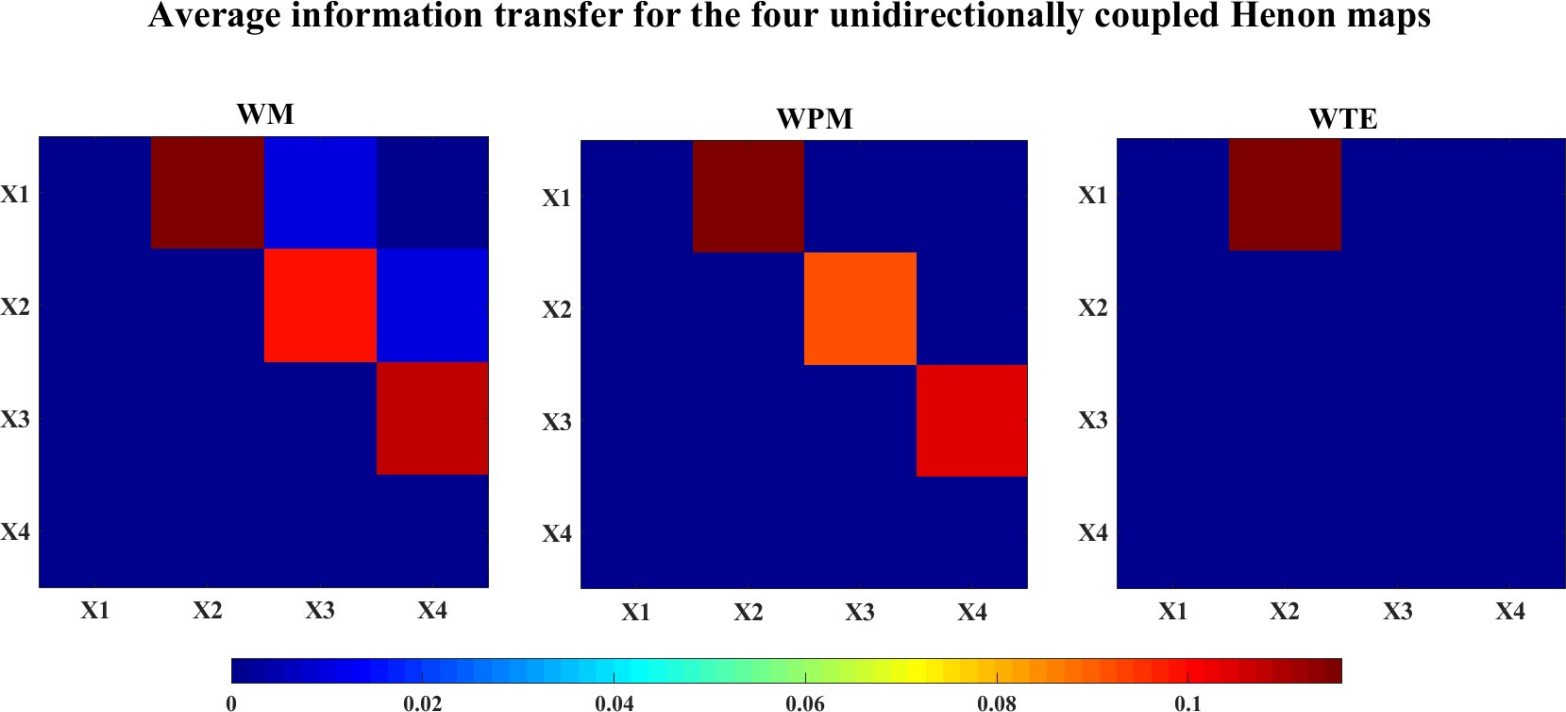
The color-map for the average information transfer between the four unidirectionally coupled Henon maps. The three color-graphs separately show the average WM (left), WPM (middle) and WTE (right) information transfer for the four unidirectionally coupled Henon maps. The direction of each lattice is read from the row channel to the column channel. In this figure, WM indicates all correct interactions from Xi−>Xj where i<j, WPM indicates only the direct directions Xi−>Xi+1,i = 1,2,3, while WTE indicates only X1->X2 and other directions are failed to be detected.

For the overall situation for the directional inference, the average information transfer values (over time scales) for the Henon maps are shown in Fig 9. In this figure, the average information transfer values are plotted by color-matrices, the correspondence between the colors and the information transfer values is shown in the color-bar. In the color-matrices, the directional inference of each lattice is from the row channel to the column channel, e.g. the (1,2)-th lattice in the color-matrix represents the average information transfer for X1 → X2. We can see that WM indicates all the correct directions of interactions, while WPM inferences only the direct interactions. In this study, WTE only indicates clear interaction from X1 → X2, although the average information transfer values for X3->X1 and X3->X2 are positive, but they are too weak compared to the strength of X1->X2, and cannot be shown in this color map.

**Fig 10:**
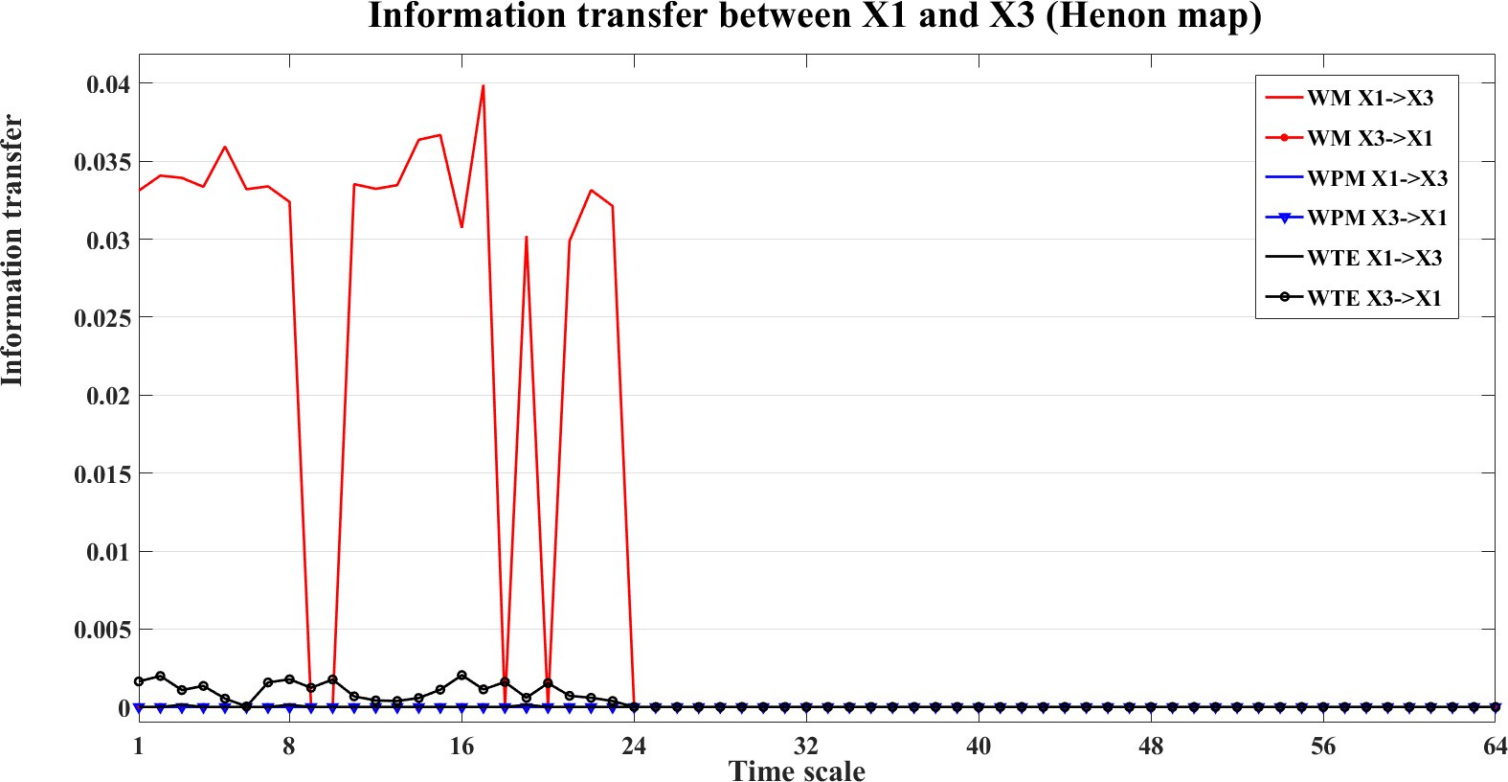
Information transfer between X1 and X3 for the four unidirectionally coupled Henon maps. In this figure, we present the bias corrected information transfer values between X1 and X3. The curves in different colors separately show the corrected WM (red), WPM (blue) and WTE (black) information transfer between X1 and X3, which are plotted against the different time scales.

**Fig 11:**
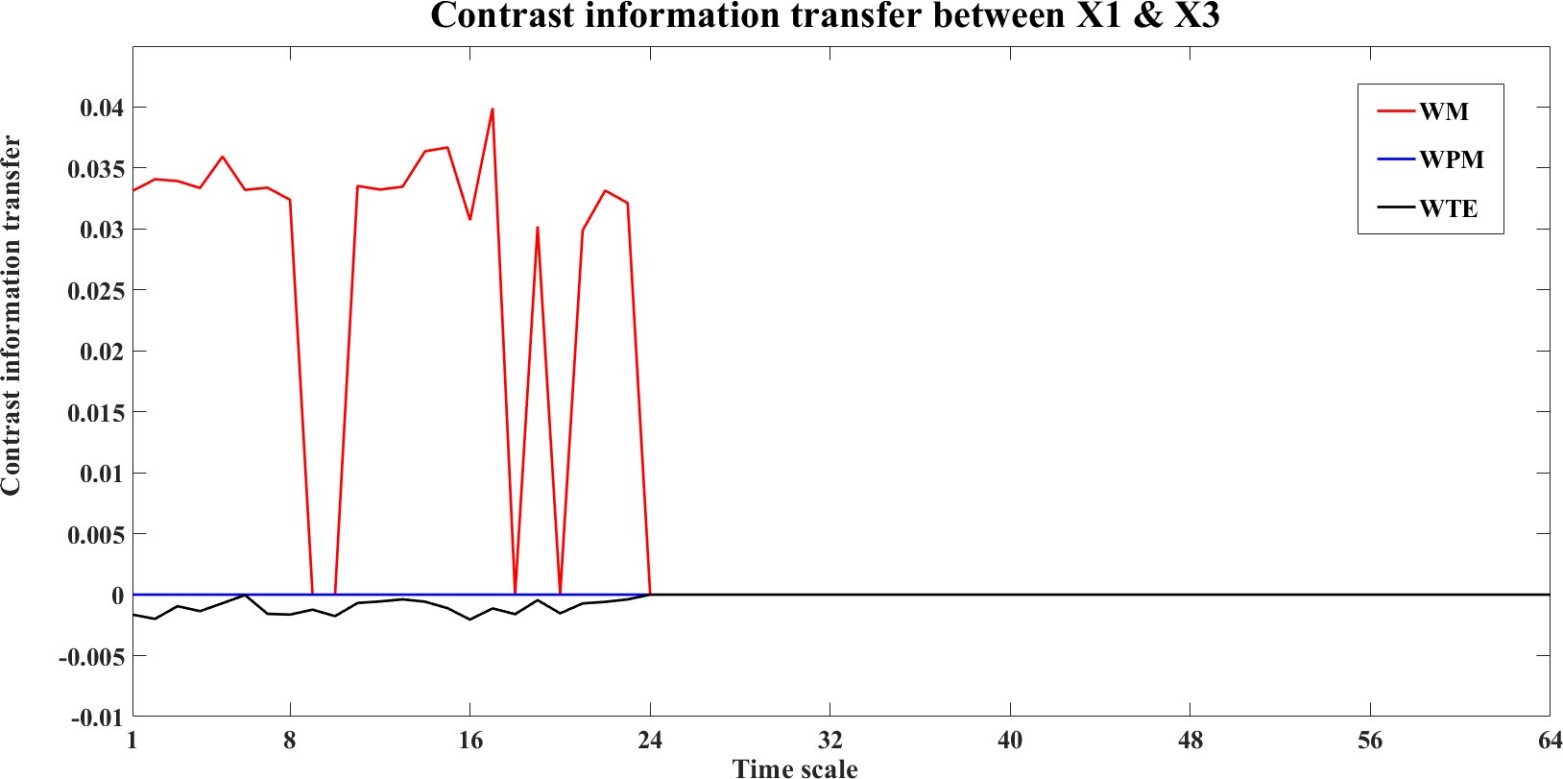
The contrast information transfer values for X1->X3 for the four unidirectionally coupled Henon maps. This figure shows the contrast WM (red), WPM (blue) and WTE (black) information transfer between X1 and X3 at different time scales *s*_*i*_ (i = 1,2,…64). WM (red) indicate the indirect information transfer from X1->X3 (positive curves), while WPM (blue) presents strictly vanished Ω_*WPM*,X1→X3_(*s*_*i*_) for all scales, which indicates no direct information transfer between X1 and X3. WTE (black) presents subtle but negative Ω_*WTE*,X1→X3_(*s*_*i*_) which indicates false information transfer from X3-> X1.

An example of the information transfer between X1 and X3 is shown in Figs 10 and 11. Fig 10 shows the bias reduced information transfer values for WM, WPM and WTE between X1 and X3. We can see from Fig 10 that WM presents positive information transfer for X1->X3 and zero information transfer for X3->X1. Since X1->X3 is an indirect direction of interaction, and WPM is a direct measure, therefore no positive information transfer is detected by WPM between X1 and X3. WTE presents false positiveness for X3->X1 but zeros information transfer for X1->X3.

The contrast information transfer values for X1->X3 (Ω_X1→X3_(*s*_*i*_),*i* = 1,…,64) are shown in Fig 11. In this figure, WM (red) indicates the indirect information transfer from X1->X3, while WPM (blue) indicates no direct information transfer between X1 and X3. WTE (black) presents negative Ω_*WTE*,X2→X3_(*s*_*i*_), which indicates false direction from X3->X1.

Also, we note that the information transfer values of WM and WPM decline to zero for large time scales. This may due to many reasons. One possible reason is that when time scale increases, the frequency and resolution decrease, details of the time series are smeared out, hence the causal interactions become too weak to be detected. The other reason is because of the characteristic correlation time of Henon maps [51–52]: if the time lag of Henon maps exceeds the characteristic cross-correlation time, the directed influence disappears [54]. The threshold of time scales may indicate the cross-correlation time of Henon maps [54]. Alternatively, it may due to the full correlation between the coupled time series that if the time series are fully correlated, the system becomes deterministic, whose values of information transfer between the wavelet coefficients become zero. The signal correlation at certain common frequencies [24, 39–43] is also be a possible reason for the vanishing causalities.

#### 3.1.3 A system of three coupled variables

The system of three coupled variables is given by the equations [50]:

$$\begin{array}{l} x_{1,t}=3.4x_{1,t-1}{\left(1-x_{1,t-1}\right)}^{2}\exp\left(-x_{1,t-1}{}^{2}\right)+0.4\varepsilon_{1,t}\\ x_{2,t}=3.4x_{2,t-1}{\left(1-x_{2,t-1}\right)}^{2}\exp\left(-x_{2,t-1}{}^{2}\right)+0.5x_{1,t-1}x_{2,t-1}+0.4\varepsilon_{2,t}\\ x_{3,t}=3.4x_{3,t-1}{\left(1-x_{3,t-1}\right)}^{2}\exp\left(-x_{3,t-1}{}^{2}\right)+0.3x_{2,t-1}+0.5x_{1,t-1}^{2}+0.4\varepsilon_{3,t} \end{array}$$ (18)

where *X*_2_→*X*_3_ is the linear interaction of the system and *X*_1_→*X*_2_ and *X*_1_→*X*_3_ are nonlinear interactions, *ε*_*i*,*t*_ (*i* = 1,…,3) are Gaussian random white noises. The data of this example can be found in S3 Dataset. The WM and WPM parameters are the same to the previous example.

**Fig 12:**
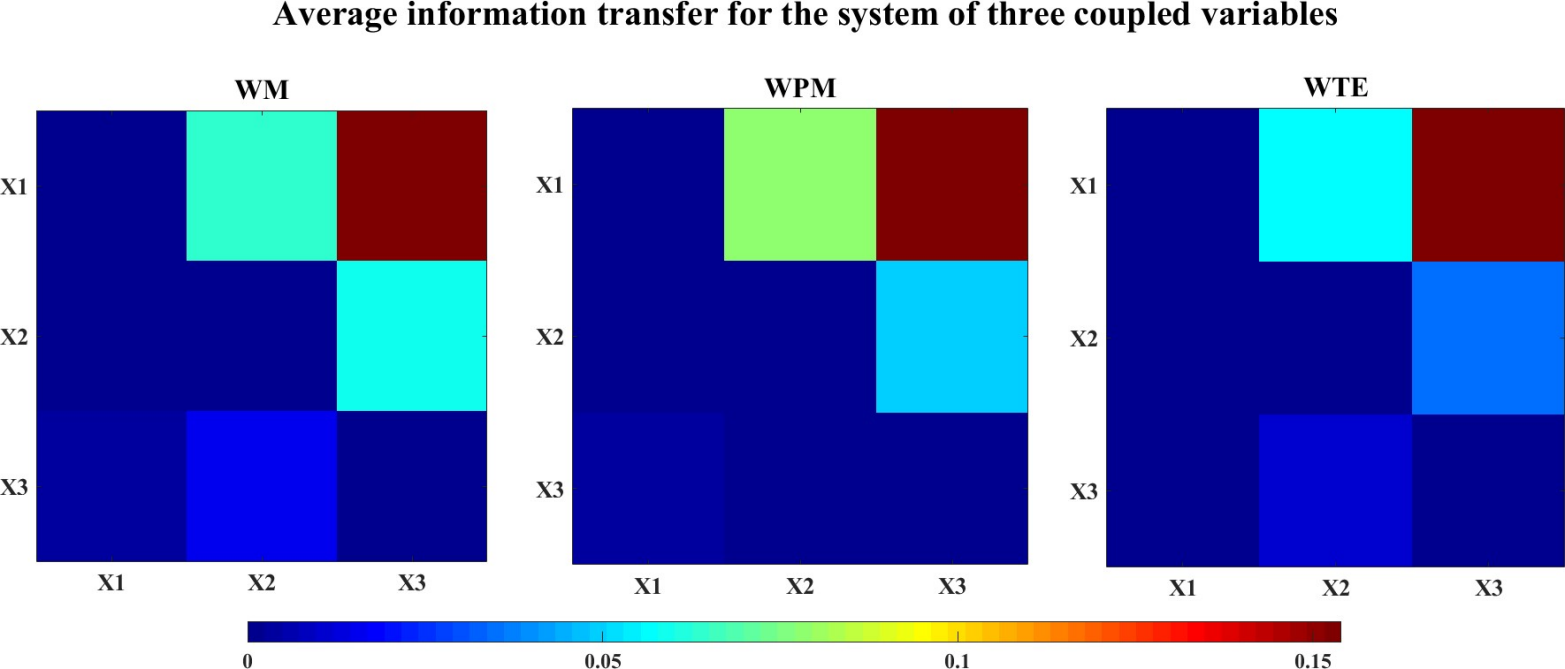
The color-map of the average information transfer values for the system of three coupled variables. The three color-maps show the average WM (left), WPM (middle) and WTE (right) values for the system of three coupled variables. The directed direction of each lattice is indicated from the row channel to the column channel. Comparing the information transfer values between opposite directions, all measures identify the correct linear (X2->X3) and nonlinear (X1->X2, X1->X3) interactions.

The average information transfer values (over all time scales) are computed for the system which are shown in Fig 12. In this figure, all measures correctly identify the linear (X2→X3) and nonlinear (X1→X2 and X1→X3) interactions.

**Fig 13:**
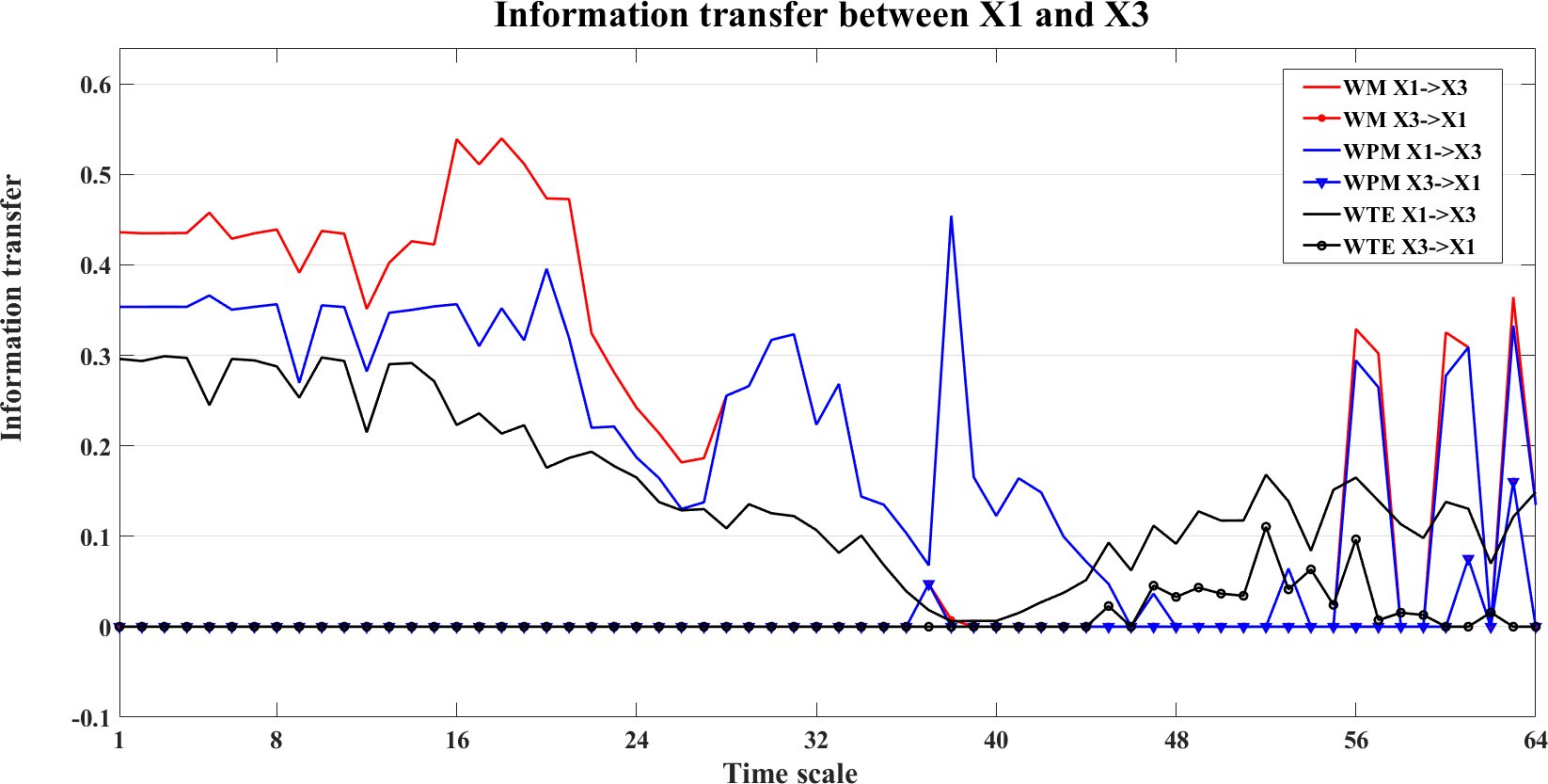
Information transfer between X1 and X3 the nonlinear interaction X1->X3. In this figure, we present the bias corrected WM (red), WPM (blue) and WTE (black) information transfer between X1 and X3 at different time scales *s*_*i*_ (i = 1,2,…64). By comparing the strength between opposite directions, all three measures indicate the correct nonlinear interaction from X1->X3.

**Fig 14:**
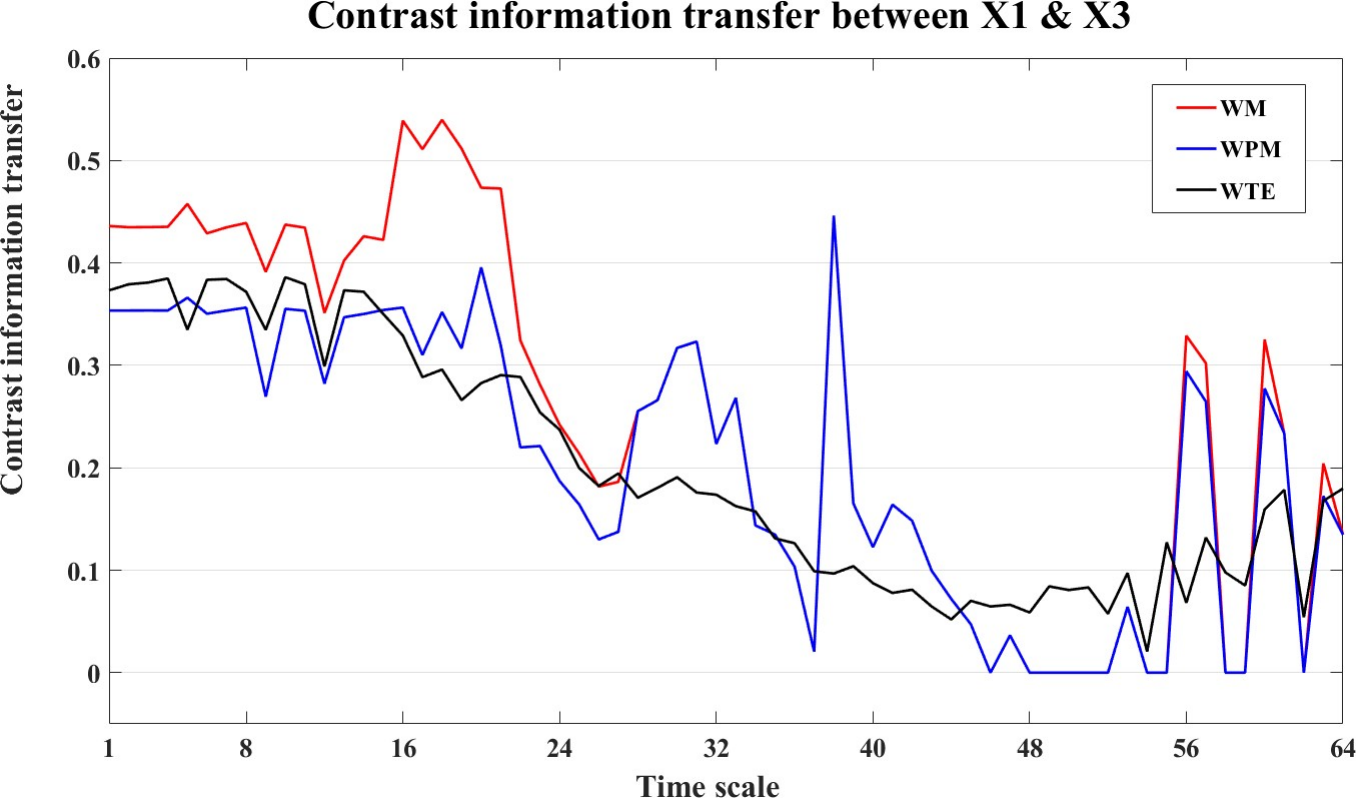
The contrast information transfer values for the nonlinear interaction X1->X3. The three curves show the contrast WM (red), WPM (blue) and WTE (black) information transfer between X1 and X3 at different time scales *s*_*i*_ (i = 1,2,…64). All three measures indicate the correct nonlinear interaction from X1->X3.

The bias corrected information transfer values are computed for each direction. The WM and WPM information transfer for the X1->X2 are similar, and all three measures present the correct interaction from X1->X2. We take the direction of X1->X3 as an example. The information transfer for both X1->X3 and X3->X1 are presented in the Fig 13. In this figure, we can see that all three measures detect the correct nonlinear interaction from X1->X3. This can also be seen from the plot of the contrast information transfer between X1 and X3 in Fig 14.

**Fig 15:**
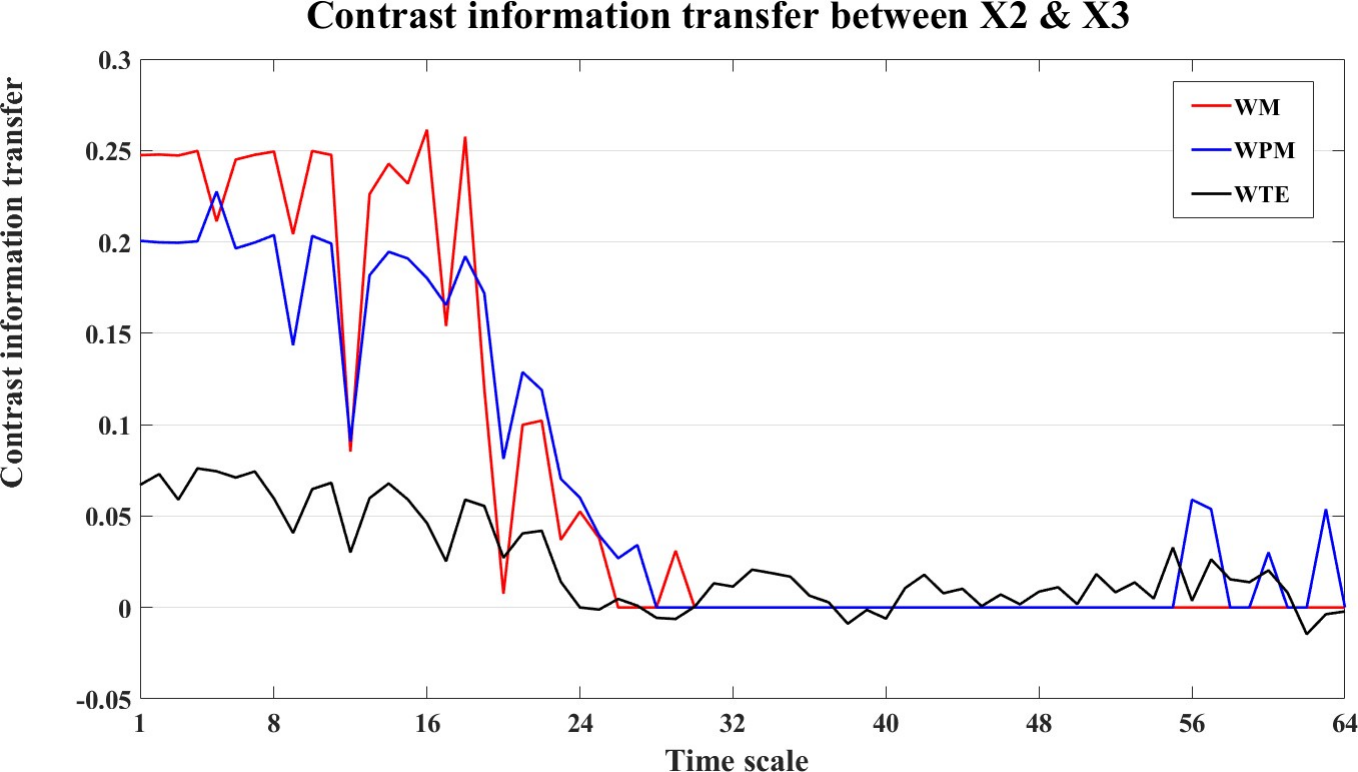
The contrast information transfer values for the linear interaction X2->X3. The three curves show the contrast WM (red), WPM (blue) and WTE (black) information transfer between X2 and X3 at different time scales *s*_*i*_ (i = 1,2,…64). All measures indicate X2->X3 at small time scale *s*_*i*_ (1≤i<30), and contrast values decline to zero at middle time scales. The contrast WM keeps zero at all larger scales, while the contrast WPM rises positive again at some large time scales. The contrast WTE fluctuates around zero level with more positive than negative values. All three measures are able to indicate the correct linear interaction from X2->X3.

For the interaction between X2 and X3, the contrast information transfer between X2 and X3 is shown in Fig 15.

#### 3.1.4 Three coupled Lorenz systems with nonlinear couplings

The three coupled Lorenz systems with nonlinear couplings *X*_1_→*X*_2_ and *X*_2_→*X*_3_ are given by the following equations [50]:

$$\begin{array}{l} {\dot{x}}_{1}=10(y_{1}-x_{1})\\ {\dot{y}}_{1}=28x_{1}-y_{1}-x_{1}z_{1}\\ {\dot{z}}_{1}=x_{1}y_{1}-\frac{8}{3}z_{1} \end{array}$$ (19)

$$\begin{array}{l} {\dot{x}}_{2}=10(y_{2}-x_{2})+k(x_{1}-x_{2})\\ {\dot{y}}_{2}=28x_{2}-y_{2}-x_{2}z_{2}\\ {\dot{z}}_{1}=x_{2}y_{2}-\frac{8}{3}z_{2} \end{array}$$ (20)

$$\begin{array}{l} {\dot{x}}_{3}=10(y_{3}-x_{3})+k(x_{2}-x_{3})\\ {\dot{y}}_{3}=28x_{3}-y_{3}-x_{3}z_{3}\\ {\dot{z}}_{3}=x_{3}y_{3}-\frac{8}{3}z_{3} \end{array}$$ (21)

where k (k = 0,1,3,5) is the coupling strength regulates the interaction from *X*_1_→*X*_2_ and *X*_2_→*X*_3_. All time series become completely synchronized when the coupling strengths k≥8. The WM and WPM parameters are the same to the previous example. The data of this example can be found in S4 Dataset.

**Fig 16:**
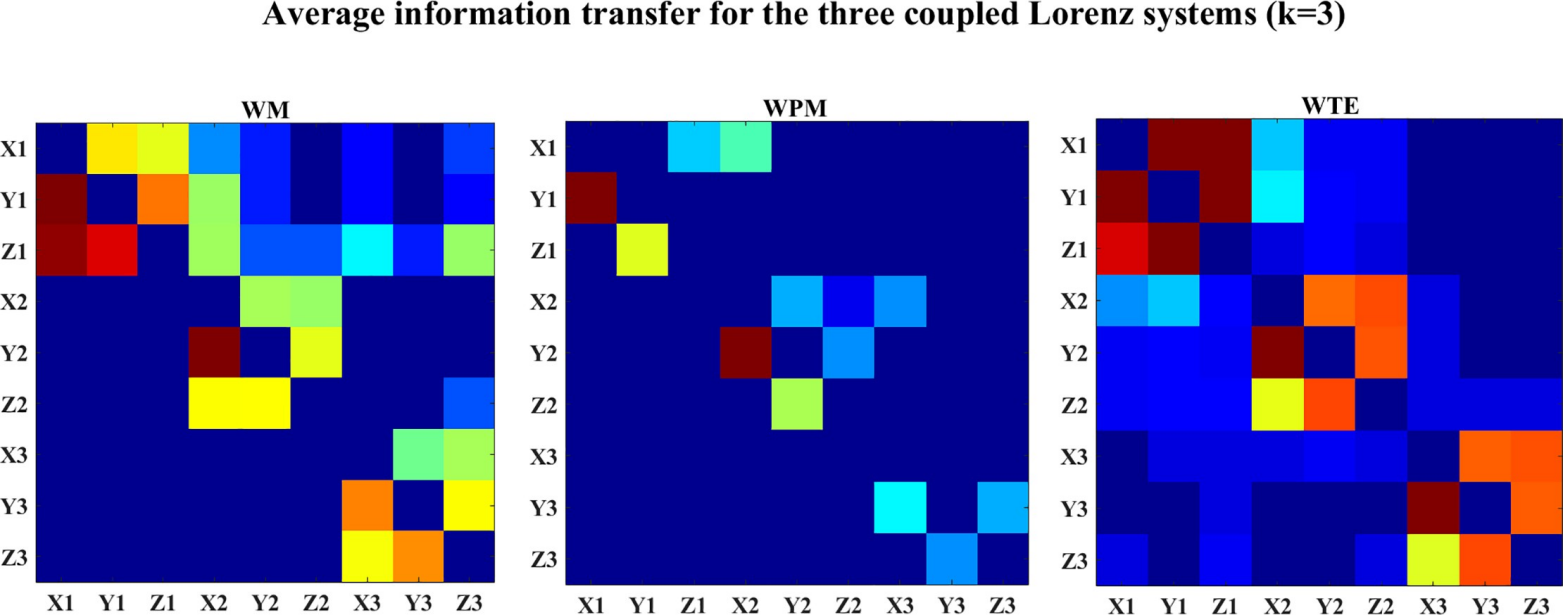
The color-map of the average information transfer for the three coupled Lorenz systems (k = 3). This figure shows the color-map for the average WM (left), WPM (middle) and WTE (right) information transfer (coupling strength k = 3) over time scales. The directed direction of each lattice is indicated from the row to the column channel. The bright diagonal blocks of WM and WPM indicate internal interaction within each system. In the WM and WPM graphs, the upper-right blocks are comparatively brighter than the lower-left blocks, which indicates the interactions between the systems. WPM indicates only direct interactions from the first to the second system and from the second to the third system, but no indirect interaction from the first to the third system. WTE presents similar strength of information transfer on both directions between coupled variables.

The average values of information transfer (over all time scales) for the Lorenz systems (c = 3) are shown in Fig 16. In this figure, both WM and WPM indicate clear interactions within each system and the cross interactions between different systems. WTE also indicates the internal and external interaction, but with less directional manner.

**Fig 17:**
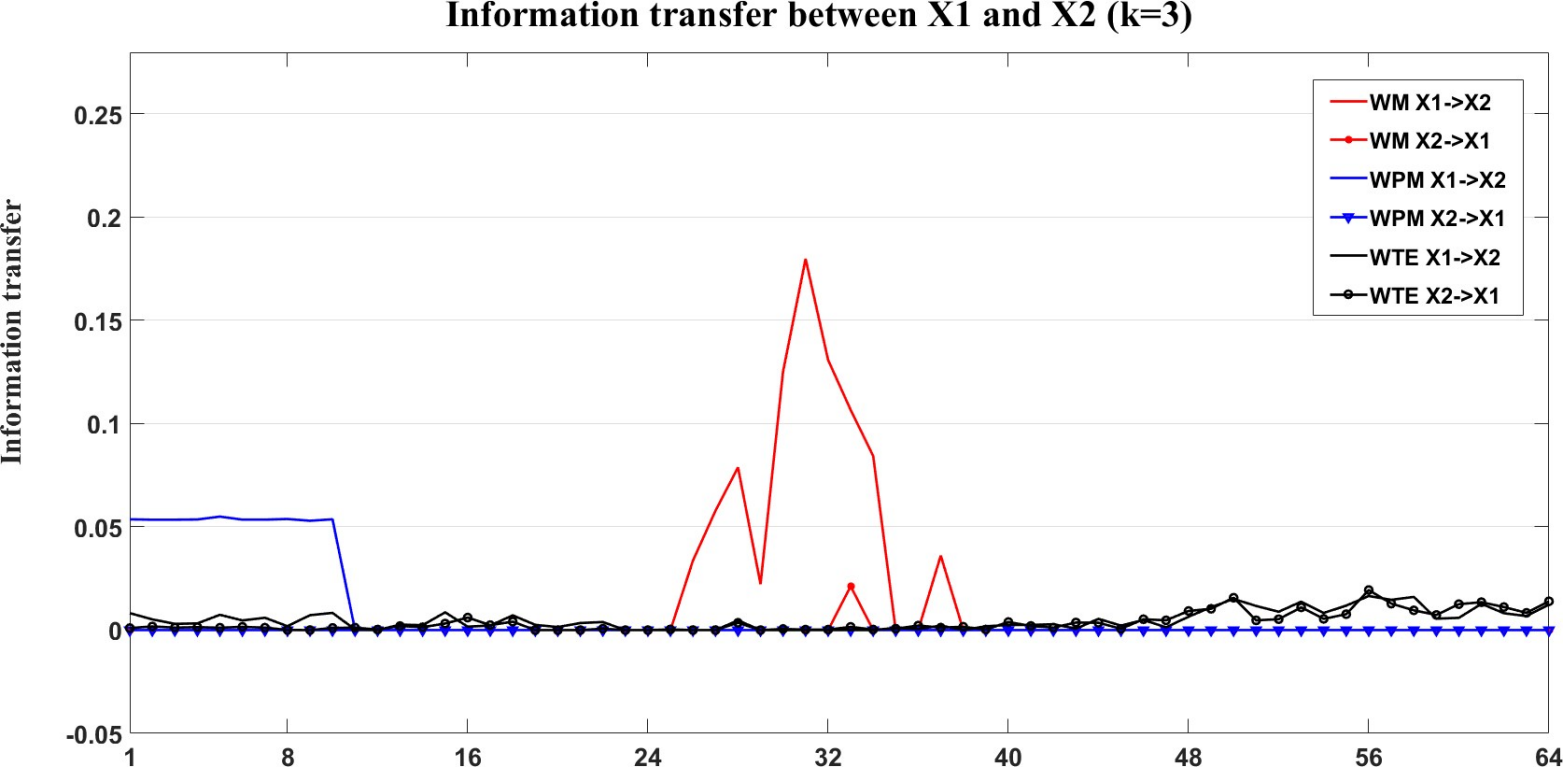
Information transfer between X1 and X2. In this figure, the bias corrected WM (red), WPM (blue) and WTE (black) information transfer between X1 and X2 are plotted at different time scales *s*_*i*_ (i = 1,2,…64). By comparing the strength of information transfer between X1 and X2, WPM identifies X1->X2 at small time scales, while WM identifies X1->X2 at middle time scales. WTE gives similar and small strength on both directions that hard to identify a clear direction for the interaction.

**Fig 18:**
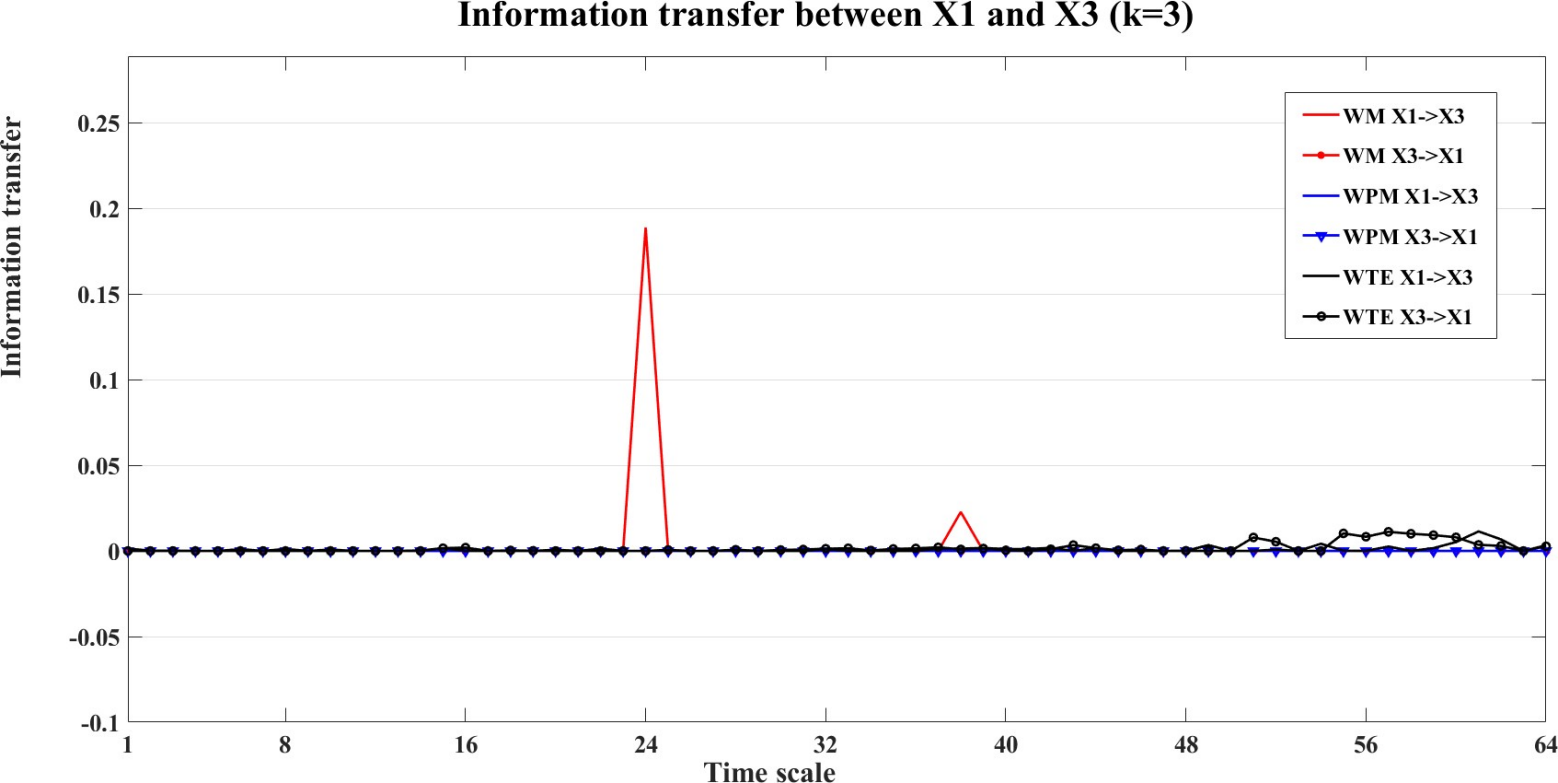
Information transfer between X1 and X3. In this figure, the bias corrected WM (red), WPM (blue) and WTE (black) information transfer between X1 and X3 are plotted at different time scales *s*_*i*_ (i = 1,2,…64). By comparing the strength between X1 and X3, only WM identifies the indirect direction of interaction from X1->X3, WPM is a direct measure that presents zero information transfer between X1 and X3. WTE presents almost zero information transfer between X1 and X3 with slightly higher X3->X1 than X1->X3.

Fig 17 shows the bias corrected information transfer between X1 and X2. In this figure, we can see that the WM indicates X1->X2 at middle time scales, while WPM indicates X1->X2 at lower time scales. WTE presents similar and subtle information transfer between X1 and X2. Similarly, the information transfer for the indirect interaction between X1 and X3 is shown in Fig 18.

**Fig 19:**
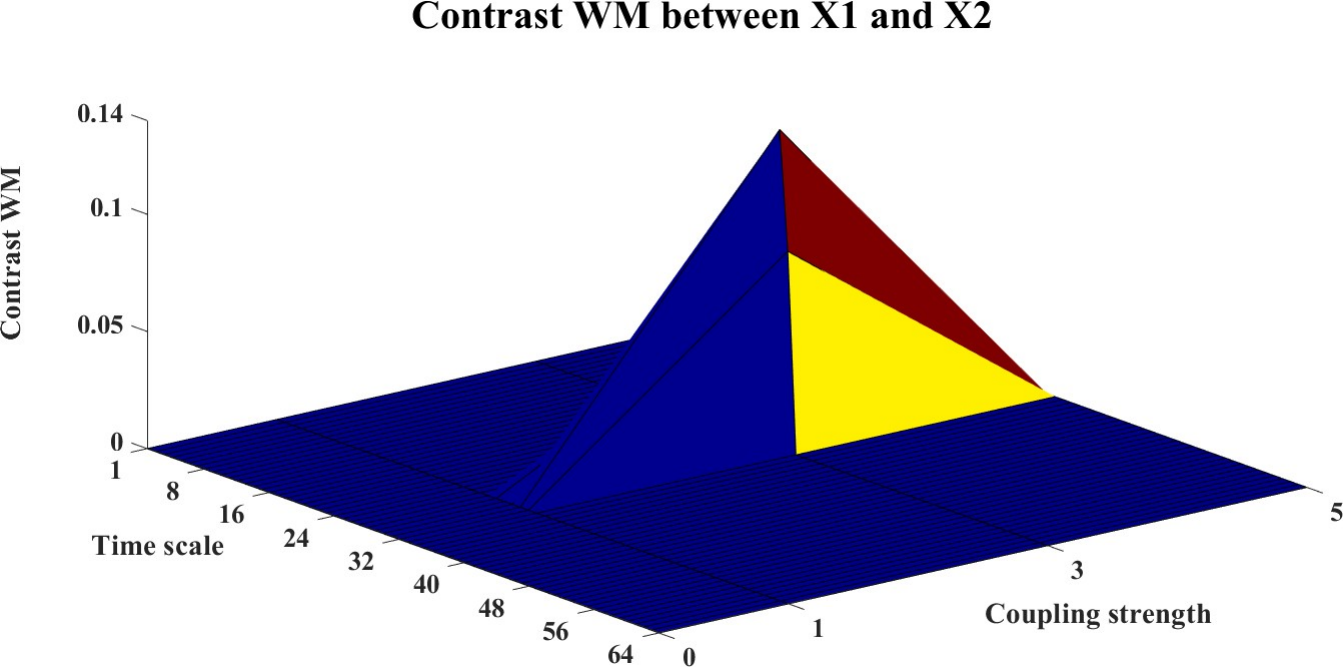
The contrast WM information transfer for X1→X2. The 3D surface presents the contrast WM information transfer (Ω_*WM*,*X*1→*X*2_(*s*_*i*_,*k*) = *WM*_*C*,*X*1→*X*2_(*s*_*i*_,*k*)−*WM*_*C*,*X*2→*X*1_(*s*_*i*_,*k*), i = 1,2,…64,k = 0,1,3,5) between X1 and X2 at different couplings and time scales. The non-negative surface indicates clear interactions from X1→X2.

**Fig 20:**
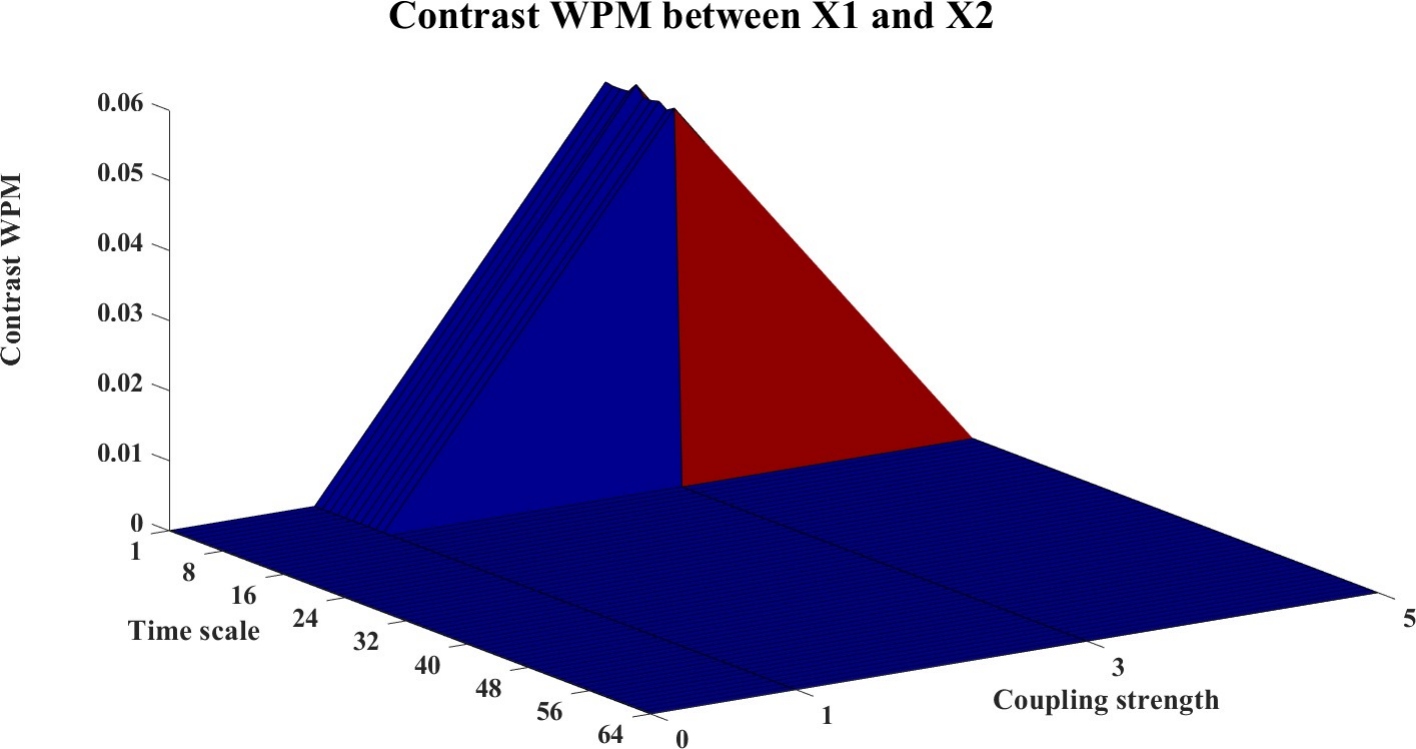
The contrast WPM information transfer for X1→X2. The 3D surface presents the contrast WPM information transfer (Ω_*WPM*,*X*1→*X*2_(*s*_*i*_,*k*) = *WPM*_*C*,*X*1→*X*2_(*s*_*i*_,*k*)−*WPM*_*C*,*X*2→*X*1_(*s*_*i*_,*k*),i = 1,2,…64,k = 0,1,3,5) between X1 and X2 at different couplings and time scales. The non-negative surface indicates clear interactions from X1→X2.

**Fig 21:**
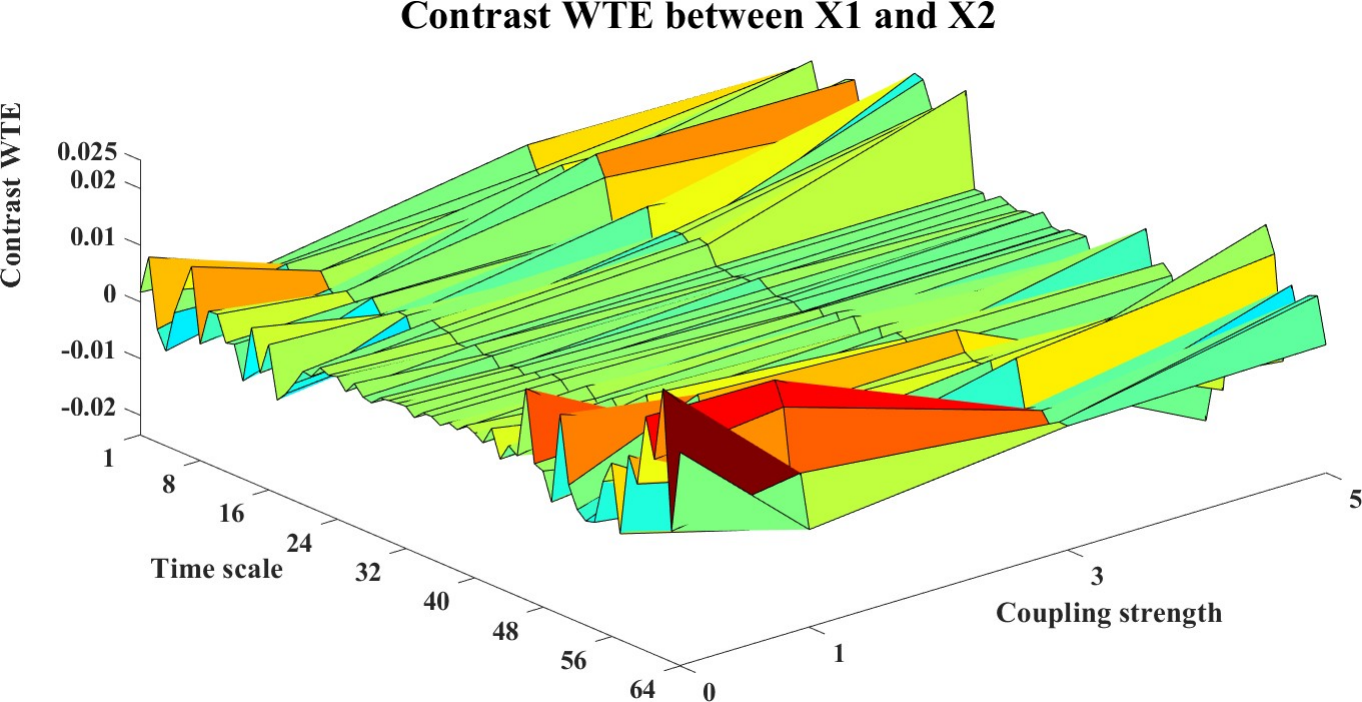
The contrast WTE information transfer for X1→X2. The 3D surface presents the contrast WTE information transfer (Ω_*WTE*,*X*1→*X*2_(*s*_*i*_,*k*) = *WTE*_*C*,*X*1→*X*2_(*s*_*i*_,*k*)−*WTE*_*C*,*X*2→*X*1_(*s*_*i*_,*k*), i = 1,2,…64,k = 0,1,3,5) between X1 and X2 at different couplings and time scales. The fluctuant surface around the zero plane fails to indicate a clear direction of the interaction.

**Fig 22:**
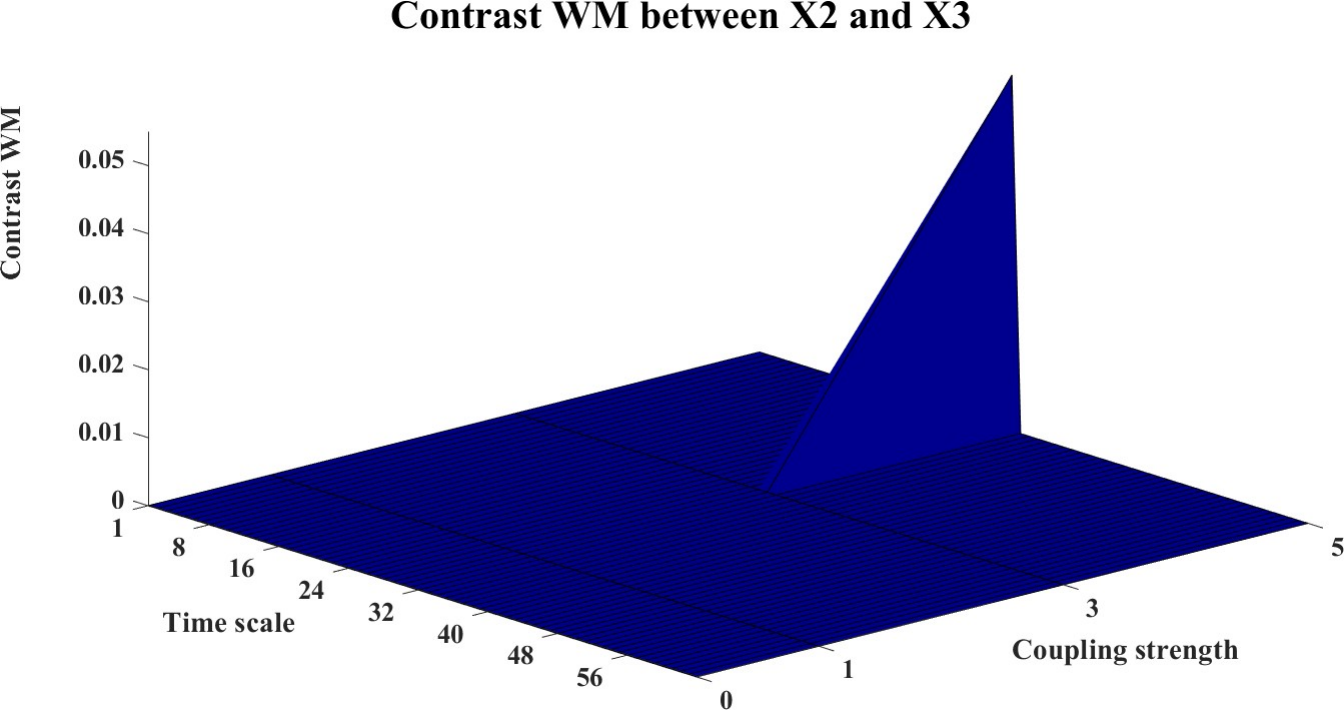
The contrast WM information transfer for X2→X3. The 3D surface presents the contrast WM (Ω_*WM*,*X*2→*X*3_(*s*_*i*_,*k*) = *WM*_*C*,*X*2→*X*3_(*s*_*i*_,*k*)−*WM*_*C*,*X*3→*X*2_(*s*_*i*_,*k*), i = 1,2,…64,k = 0,1,3,5) between X2 and X3 at different couplings and time scales. The positive ridge of the surface indicates the directed interaction from X2→X3.

**Fig 23:**
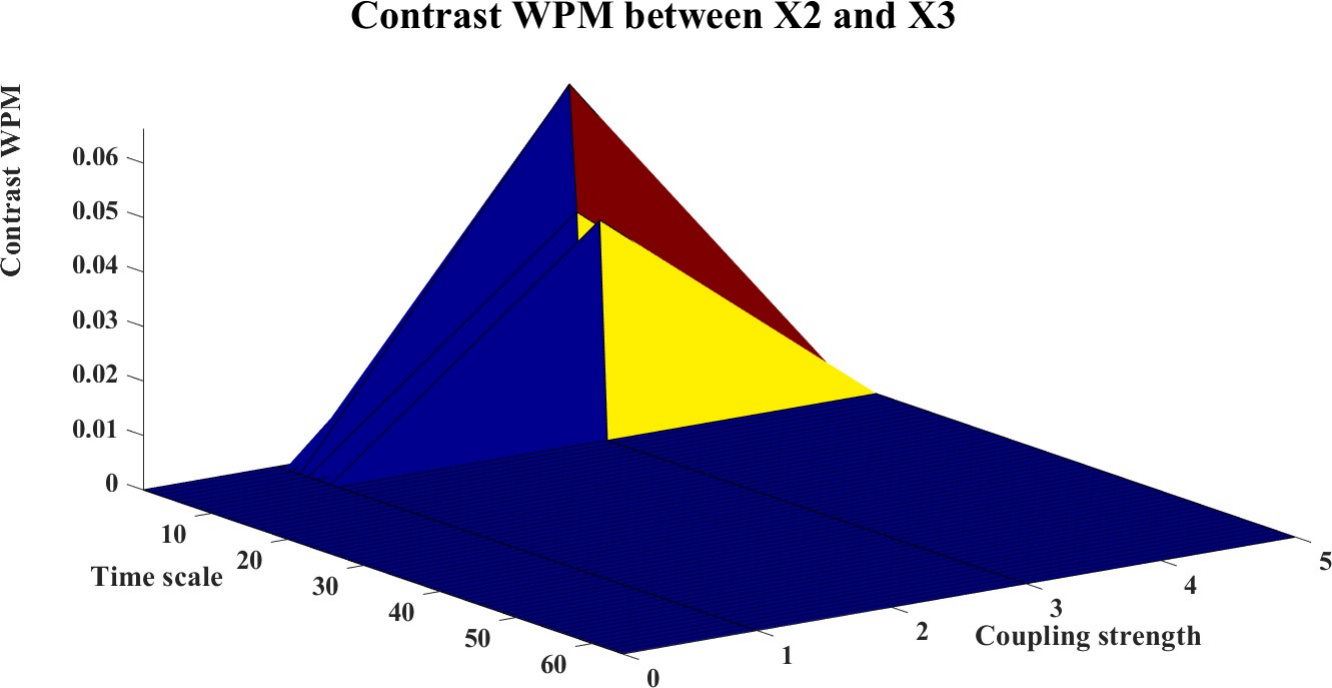
The contrast WPM information transfer for X2→X3. The 3D surface presents the contrast WPM (Ω_*WPM*,*X*2→*X*3_(*s*_*i*_,*k*) = *WPM*_*C*,*X*2→*X*3_(*s*_*i*_,*k*)−*WPM*_*C*,*X*3→*X*2_(*s*_*i*_,*k*),i = 1,2,…64,k = 0,1,3,5) between X2 and X3 at different couplings and time scales. The positive surface indicates the directed information flows from X2→X3.

**Fig 24:**
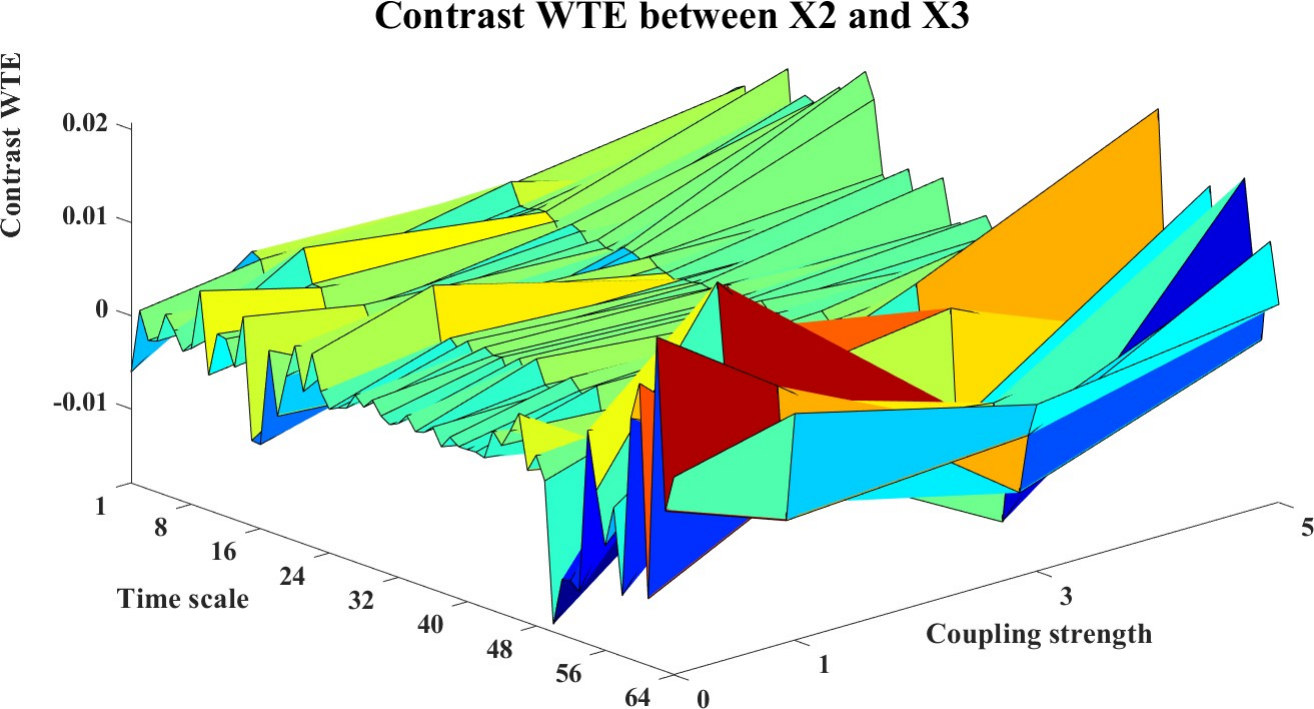
The contrast WTE information transfer for X2→X3. The 3D surface presents the contrast WTE (Ω_*WTE*,*X*2→*X*3_(*s*_*i*_,*k*) = *WTE*_*C*,*X*2→*X*3_(*s*_*i*_,*k*)−*WTE*_*C*,*X*3→*X*2_(*s*_*i*_,*k*),i = 1,2,…64,k = 0,1,3,5) for X2→X3 at different couplings and time scales. The fluctuant sign of the surface indicates no clear direction of interactions.

The contrast information transfers between the systems are shown in Figs 19–24. Figs 19–21 present the contrast information transfer between X1 and X2, in these figures, we see that both WM and WPM give clear positive information transfers for X1->X2, while WTE presents fluctuate information transfers between X1 and X2. Similarly, Figs 22–24 present the contrast information transfer between X2 and X3, in these figures, both WM and WPM present the positive contrast which indicate the correct directional inference for X2->X3, the WTE again gives biased contrast that cannot indicate a clear direction.

### 3.2 Real world time series

In this section, we use two real-world data examples to demonstrate the analysis of WM and WPM. The examples include a set of EEG data measured from experiments and a financial data set observed from real market.

#### 3.2.1 The reading experiment

The reading experiment is comprised of a reader and a listener whose EEG data are measured when the reader is reading a short story to the listener. This experiment has been reported in [26, 38] for information flow test. The EEG data is made up of 10 channels for each participant, which are measured from 10 international standard electrodes [26, 38] at 100Hz frequency. The set of EEG time series are typical nonlinear and non-stationary [2–5]. The reader and the listener together form a “driver-responder” system. Here, we use WM and WPM to test the information transfer for the EEG data. The data of the reading experiment can be found in S5 Dataset.

To view the instantaneous dynamics of the system, the entire data is split into equal-space time windows of 4 seconds [26, 38]. The entire data contains 30 such time windows, we use intermediate 20 consecutive time windows (from the 6^th^ to the 25^th^ windows) to demonstrate the analysis. WM and WPM are supposed to detect the directed interaction from the reader to the listener [26, 38].

**Fig 25:**
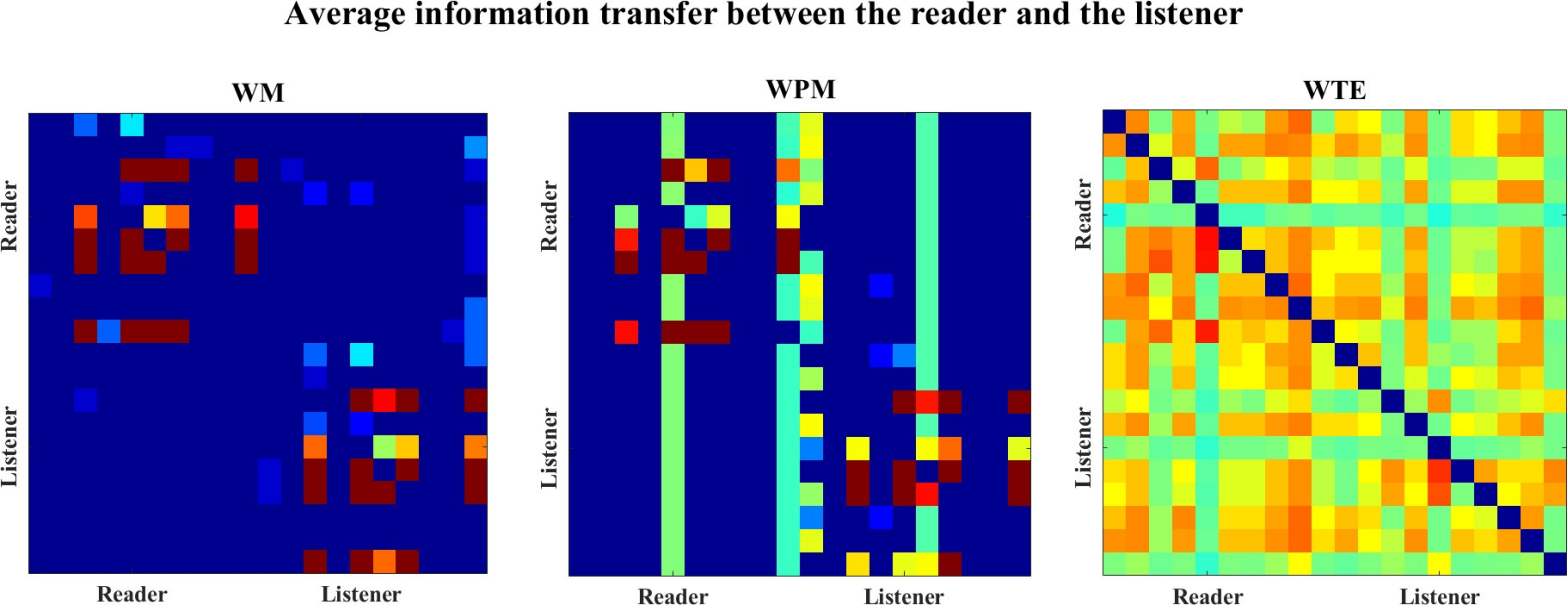
The color-map for the average information transfer between the reader and the listener. The color-graphs separately show the average WM (left), WPM (middle) and WTE (right) values for the 20 channel EEGs. For each lattice the directional inference is read from the row channel to the column channel. In the color-matrices, the diagonal blocks present the “intra-brain” information transfers within each participant, while the off-diagonal blocks present the “cross-brain” interactions across different participants.

Fig 25 shows the average values of information transfer (over windows and scales) for the 20 channel EEGs of reader and the listener. The color lattices indicate the magnitudes of the average information transfer values, whose direction is read from the row channel to the column channel. In this figure, the 20x20 color-matrix are divided into two 10x10 diagonal blocks presenting “intra-brain” interactions within the participants and two 10x10 off-diagonal blocks presenting the “cross-brain” interactions between different participants. Here, both WM and WPM indicate information transfer from the reader to the listener, because the upper-right block (Reader->Listener) is bit brighter than the lower-right block (Listener->Reader). The color-matrix of WTE is nearly symmetric, which is hard to detect a clear direction.

**Fig 26:**
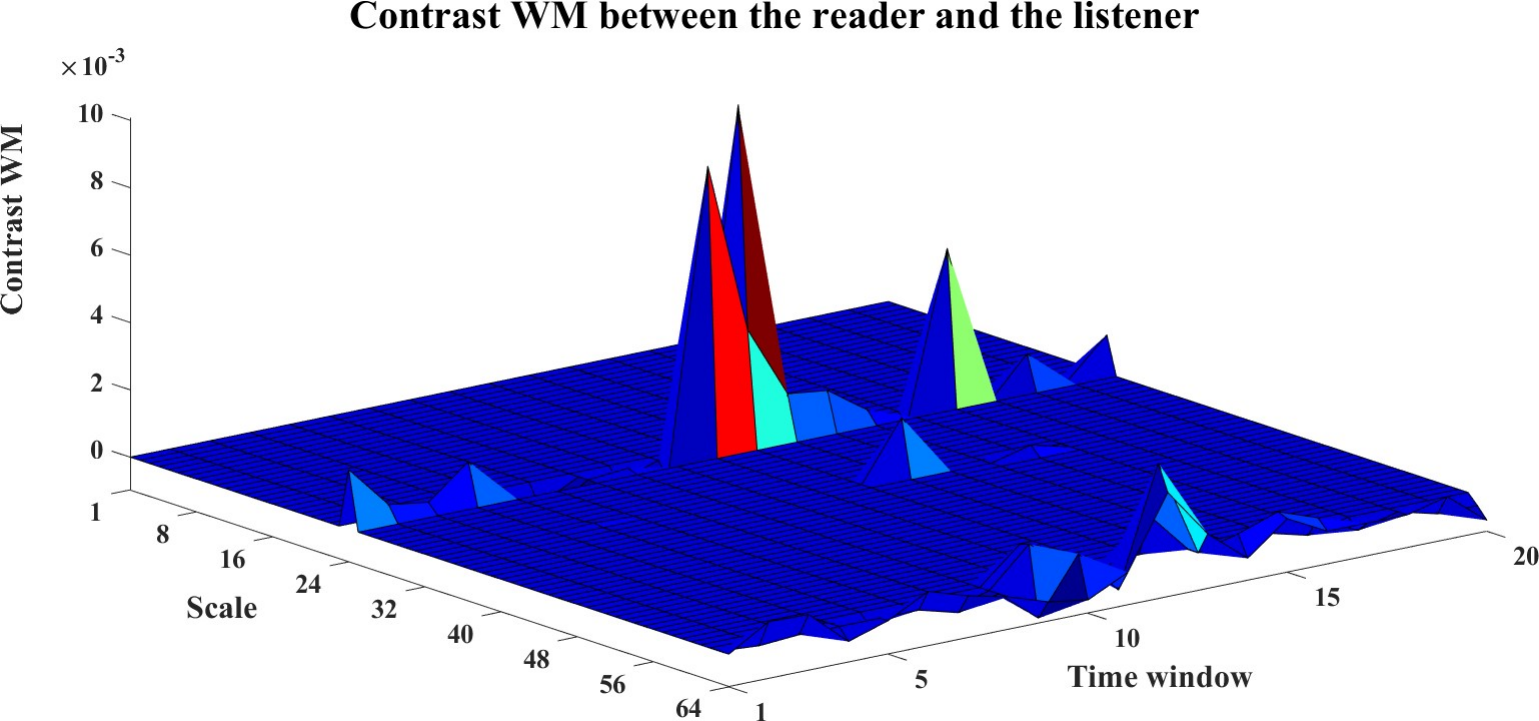
The contrast WM for Reader->Listener. The 3D surface presents the contrast WM for Reader→Listener at different scales and time windows (Ω_*WM*,*R*→*L*_(*s*_*i*_,*w*) = *WM*_*C*,*R*→*L*_(*s*_*i*_,*w*)−*WM*_*C*,*R*→*L*_(*s*_*i*_,*w*),i = 1,2,…64,w = 1,2,…,20).

**Fig 27:**
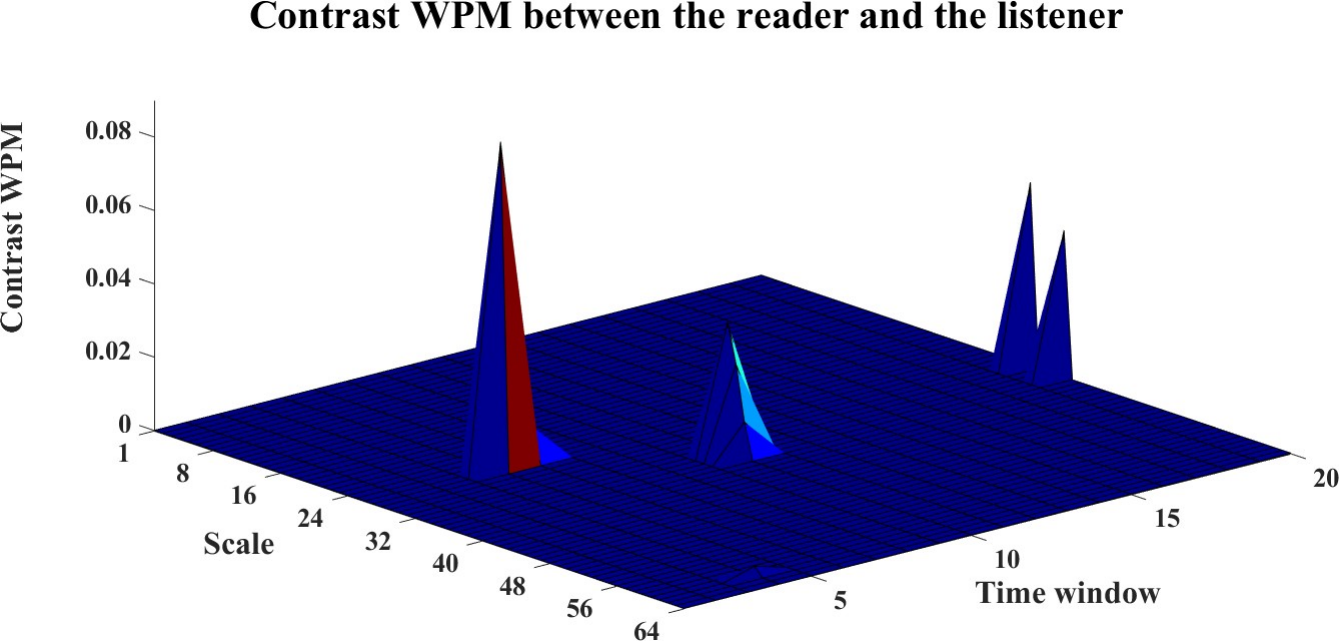
The contrast WPM for Reader->Listener. The 3D surface presents the contrast WPM for Reader→Listener at different scales and time windows (Ω_*WPM*,*R*→*L*_(*s*_*i*_,*w*) = *WPM*_*C*,*R*→*L*_(*s*_*i*_,*w*)−*WPM*_*C*,*R*→*L*_(*s*_*i*_,*w*), i = 1,2,…64,w = 1,2,…,20).

**Fig 28:**
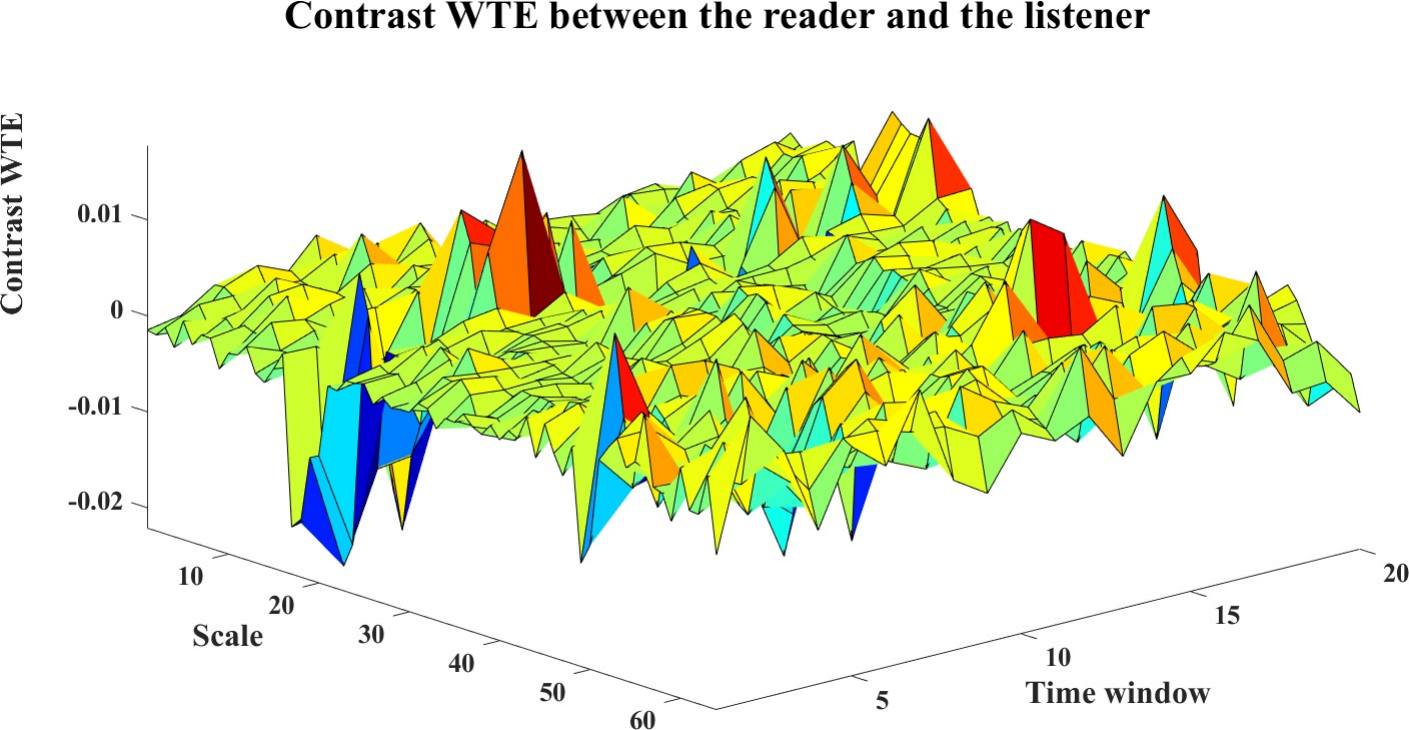
The contrast WTE for Reader->Listener. The 3D surface presents the contrast WTE for Reader→Listener at different scales and time windows (Ω_*WTE*,*R*→*L*_(*s*_*i*_,*w*) = *WTE*_*C*,*R*→*L*_(*s*_*i*_)−*WTE*_*C*,*R*→*L*_(*s*_*i*_), i = 1,2,…64,w = 1,2,…,20).

To analyze the directed interaction between the reader and the listener, the contrast information transfers for Reader→Listener are plotted in Figs 26–28. We can see from Figs 26 and 27, both WM and WPM present positive ridges for the contrast information transfer, which indicate dominant information transfer from Reader→Listener. The WTE results as shown in Fig 28 present fluctuant surface around the zero plane, which is hard to indicate a clear direction.

#### 3.2.2 Fixed incomes

The fixed incomes data are composed of 10 sovereign bond futures issued by different countries and with different maturities. The labels of these futures are listed as follows: CAN10 (Canadian, 10 years maturity), GER10 (German, 10 years maturity), GER5 (German, 5 years maturity), GER2 (German, 2 years maturity), US15 (US, 15 years maturity), US25 (US, 25 years maturity), US10 (US, 10 years maturity), US5 (US, 5 years maturity), US2 (US, 2 years maturity). The data is extracted from E-Signal on November 13^th^ at 12:51 and goes back to October 18^th^ at 13:00, on a minute-by-minute basis and in the unit of US dollars. We use a synchronized segment of the data to demonstrate the analysis. The data of the fixed incomes can be found in S6 Dataset.

**Fig 29:**
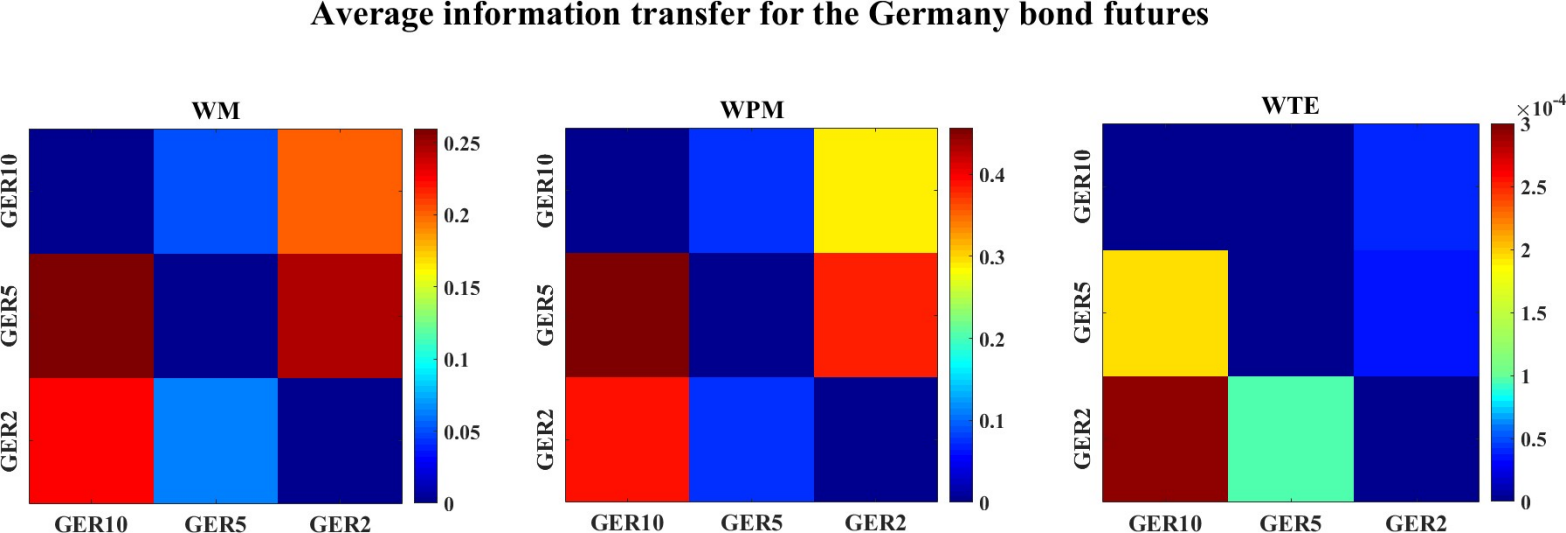
The color-map for the average information transfer between the Germany bond futures. The three color-graphs present the color-map of the average WM (left), WPM (middle) and WTE (right) information transfer between the Germany bond futures. The direction of information flow is read from the row channel to the column channel for each lattice.

Financial time series are often nonlinear and non-stationary [6], we use WM and WPM to analyze the inter dynamics between different bond futures. Fig 29 shows the average information transfer (over time scales) for the Germany bond futures with different maturities. Both WM and WPM indicate the information transfer from GER5->GER10, GER5->GER2, and GER2->GER10. WTE also indicates GER5->GER10 and GER2->GER10, but with another direction from GER2->GER5.

**Fig 30:**
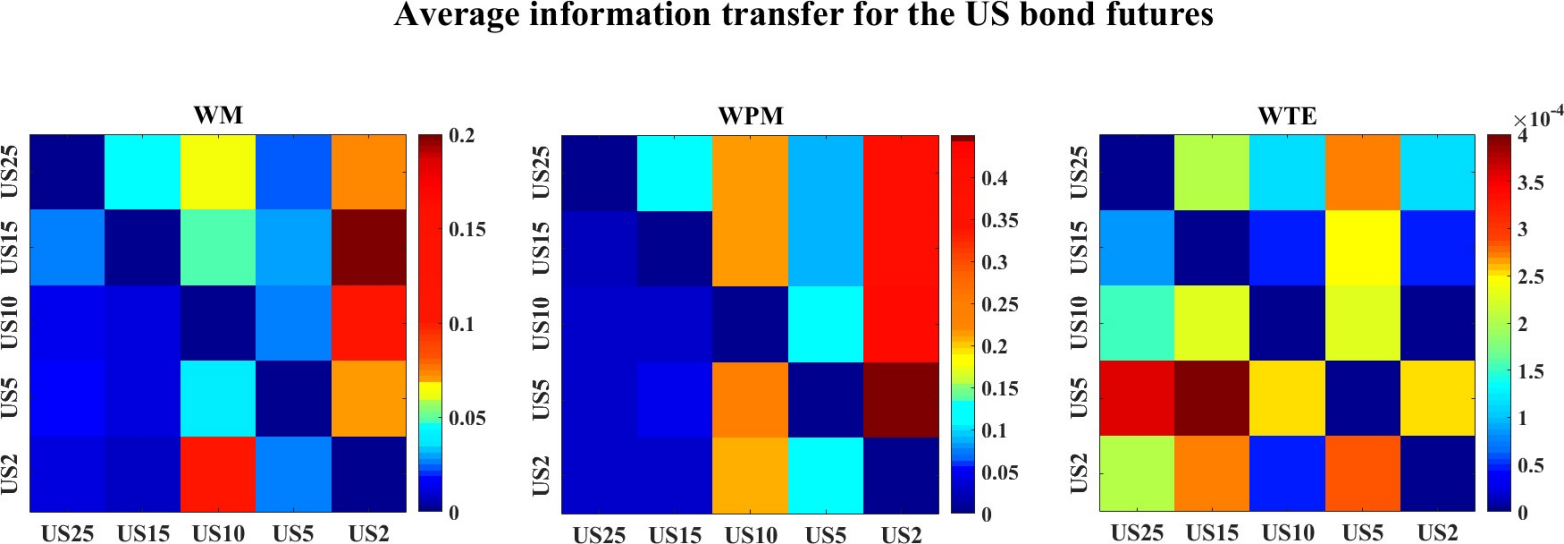
The color-map for the average information transfer values of the US bond futures. The three color-graphs present the color-map for the average WM (left), WPM (middle) and WTE (right) information transfer for the US bond futures. The direction of interaction is read from the row channel to the column channel for each lattice.

Fig 30 shows the average information transfer values (over time scales) for the US bond futures. Both WM and WPM indicate the long-year US bond futures influence the short-year US bond futures such as US10 and US2. WTE identifies strong influence from US5 to US25 and US15, but it fails to inference the other directions.

**Fig 31:**
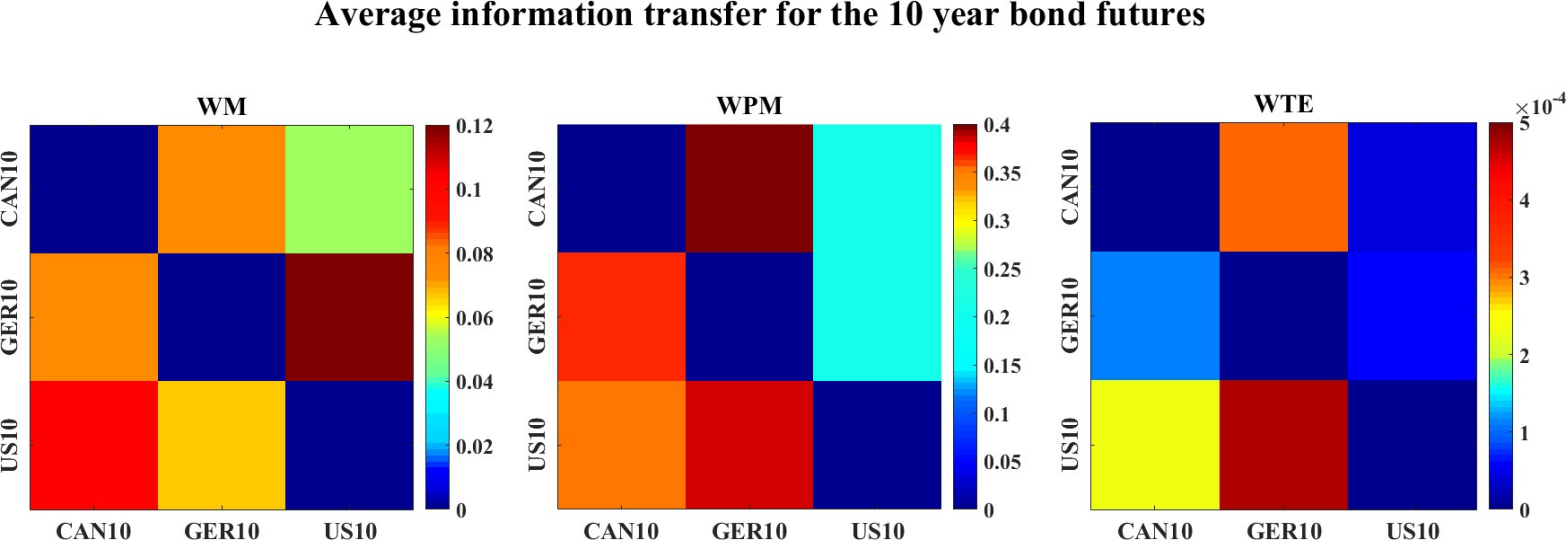
The color-map for the average information transfer values of the 10-year bond futures. The three color-graphs present the color-map for the average WM (left), WPM (middle) and WTE (right) values for the 10-year bond futures. The direction of interaction is read from the row channel to the column channel for each lattice.

Fig 31 plots the average information transfer values (over time scales) between all 10-year bond futures issued by different countries. All three measures indicate US10→CAN10, WM also indicates GER10->US10, while WPM and WTE indicate CAN10->GER10 and US10→GER10.

**Fig 32:**
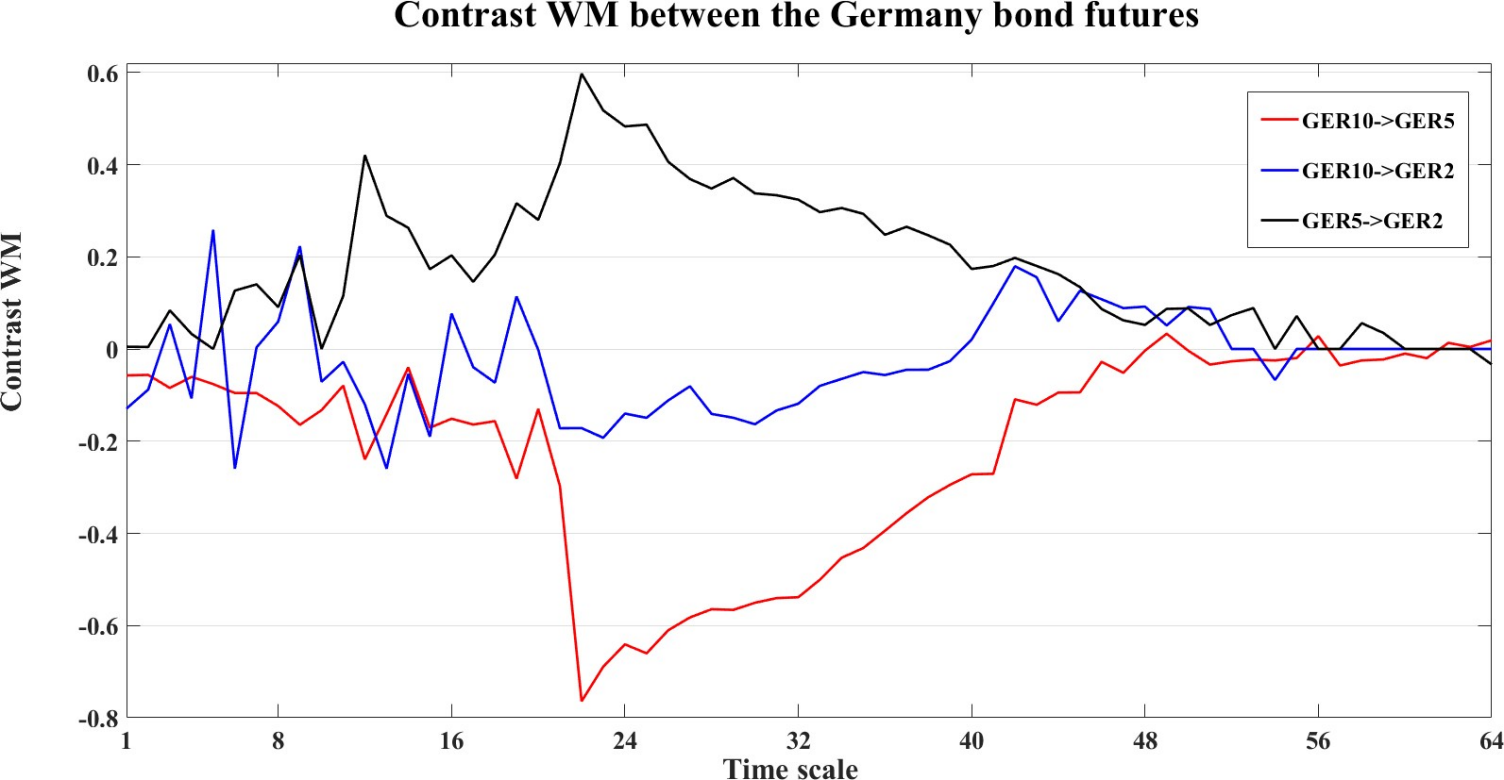
The contrast WM information transfer values for the Germany bond futures. The line graphs show the contrast WM values between the Germany bond futures at different time scales *s*_*i*_ (i = 1,2,…64).

**Fig 33:**
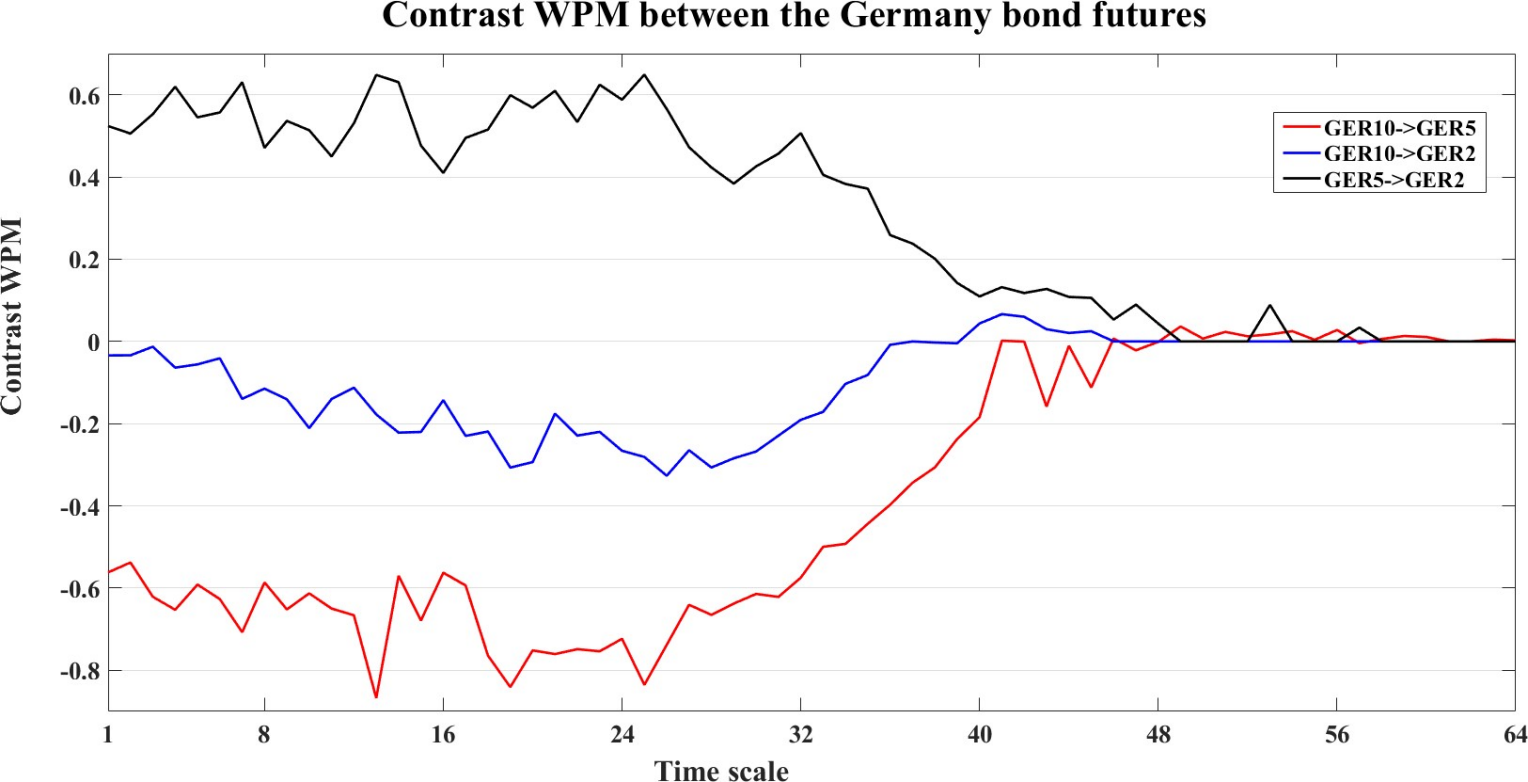
The contrast WPM information transfer values for the Germany bond futures. The line graphs show the contrast WPM values between the Germany bond futures at different time scales *s*_*i*_ (i = 1,2,…64).

**Fig 34:**
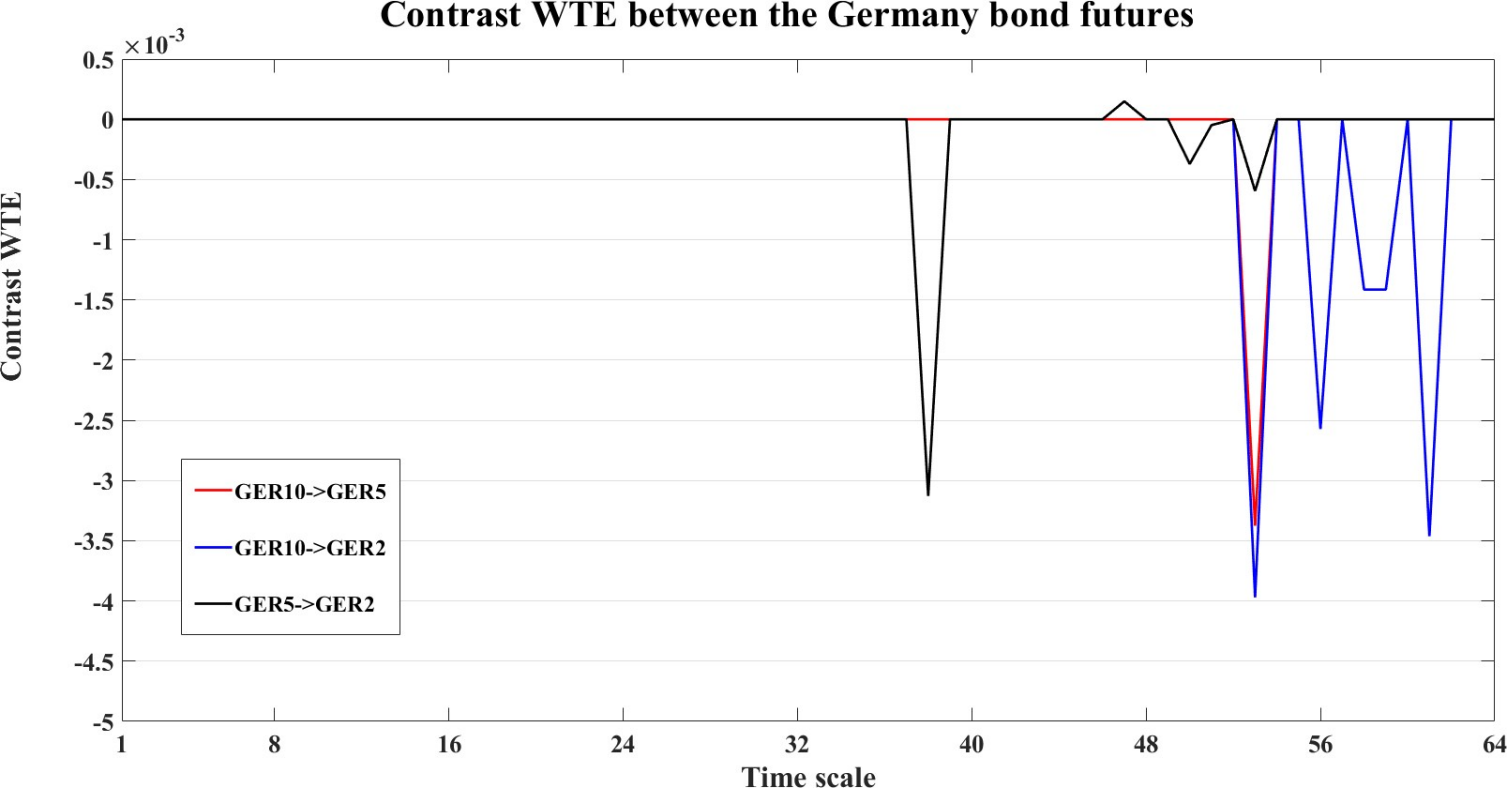
The contrast WTE information transfer values for the Germany bond futures. The line graphs show the contrast WTE values between the Germany bond futures at different time scale *s*_*i*_ (i = 1,2,…64).

To study the dynamics at different time scales, we also compute the contrast information transfer for the three measures, an example of the Germany bond futures is shown in Figs 32–34.

In Fig 32, WM indicates clear interactions from GER5->GER2 (black) and GER5->GER10 (red), and fluctuant interaction between GER10 and GER2 (blue). WPM (Fig 33) also indicates clear interaction from GER5->GER2 (black) and GER5->GER10 (red), and also GER2->GER10 (blue). WTE (Fig 34) indicates GER5->GER10 (red), GER2->GER10 (blue) and GER2->GER5 (black).

**Fig 35:**
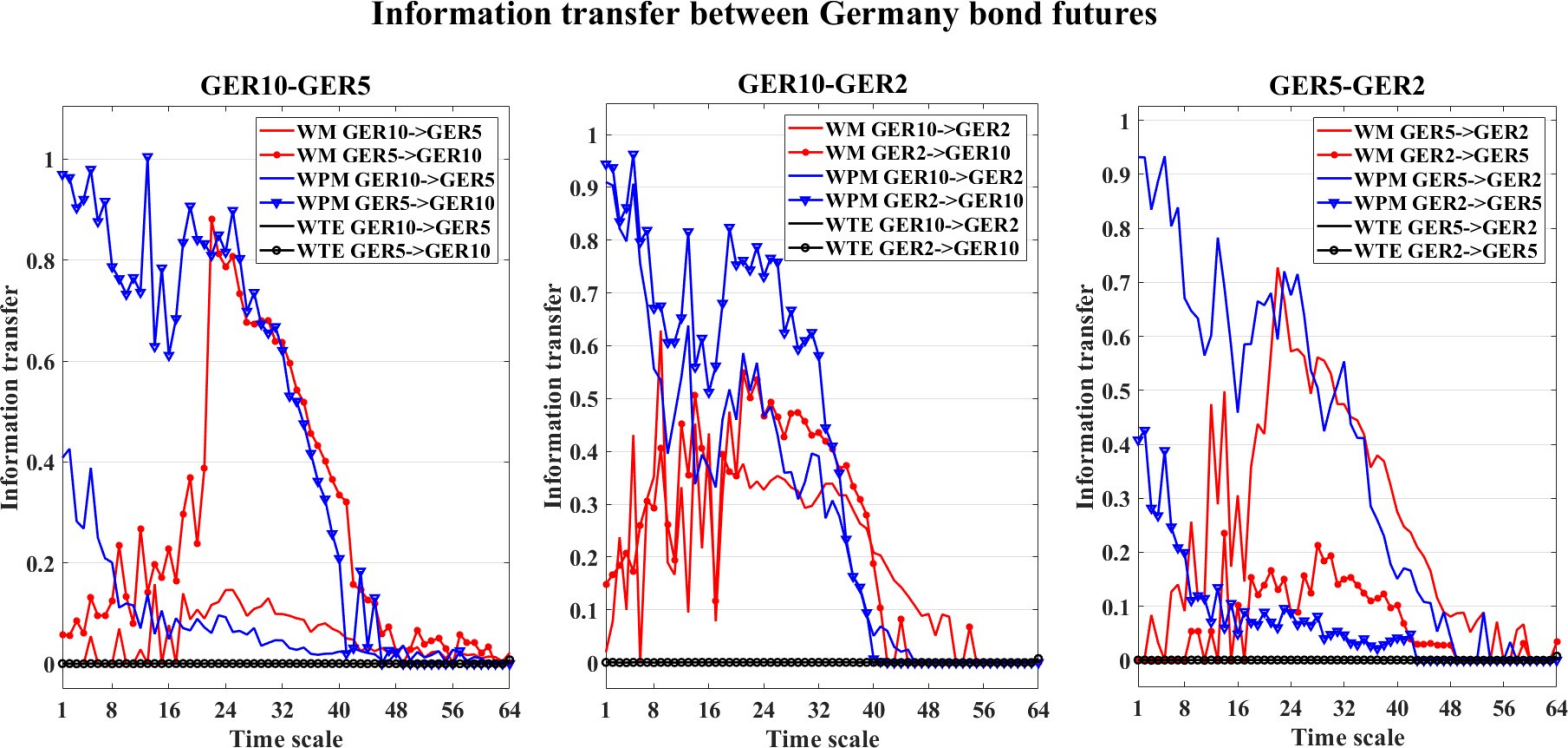
Information transfer between the Germany bond futures. In this figure, the three graphs show the information transfer between the three Germany bond futures (GER10, GER5, GER2). In each graph, the WM (red), WPM (blue) and WTE (black) information transfer values are plotted against different time scales *s*_*i*_, i = 1,2,…64.

We also examine the information transfer for each independent direction. An example of the information transfer between Germany bond futures is shown in Fig 35. In this figure, the three graphs separately show the directed information transfer between the three Germany bond futures. In these graphs, we can see that both WM and WPM give higher information transfer values for GER5->GER10, WTE presents some of the positiveness for GER5->GER10, but the strength of WTE is quite subtle that almost vanish. For the interaction between GER10 and GER2, both WM and WPM indicate dominant information flows from GER2->GER10 at middle time scales, again WTE presents subtle information transfers that approximately vanish. In the third graph, both WM and WPM show dominant information flow from GER5->GER2, while WTE fails to identify the interactions.

The overall results of the directed interaction for the bond futures agree with the outcomes found by MIME [55]. The difference is that the WM and WPM can interpret the directed interaction at multi-scales. Since the scales are related to the frequencies, one can use these multi-scale measures to specify the interactions or time series correlation at specific scales or frequencies, the multi-scale interpretation of information transfer may help one to find more information from given datasets.

## Discussion

In this paper, we propose two multi-scale information transfer measures, namely the WM and WPM, which are extended from conditional mutual information measures by using MORLET wavelet. By a series of simulation studies, the two measures are proved to be efficient and accurate in directional inference, which are computational efficient and outperform the wavelet extension of transfer entropy (WTE) in various situations. Particularly, the two measures are very useful in real-world data analysis.

The two proposed measures have many good advantages. By using wavelet, the proposed measures are able to inference the directed interactions at multi-scales. This not only helps to discover more information between coupled time series, but also solves the problem for non-stationary and discontinuous data analysis [24, 39–43]. One reason that we use MORLET wavelet to do the measure extensions is that MORLET wavelet is believed to be closely related to human perception and has vital applications to medicine [39–43], which may have wide-applications in medicine and biological data analysis e.g. EEG data analysis. The other reason is that M. Lungarella and A. Pitti have successfully introduced MORLET wavelet to Transfer Entropy, which is proved to be feasible in information transfer detection [24] of non-stationary and discontinuous data analysis. Since real-world time series are sometimes non-stationary and discontinuous, it is necessary to have this wavelet-extension that suits for practical data analysis.

Due to the basis of conditional mutual information measures, the proposed multi-scale measures can have wide-applications to various data models. Information transfer measures are a type of very useful measures. Although we note that information transfer is a different concept from that of the causal effect [31–34], but the efficiency of directional inference and the model-free advantage of the information transfer measures guarantee their wide-applications in various types of data analysis particularly the real-world data analysis [26].

Transfer entropy (TE) is a fundamental information transfer measure, which is a good prototype for other derivative information measures. The work by M. Lungarella and A. Pitti [24] shows the wavelet derivation of TE for non-stationary and discontinuous data analysis. However, WTE uses uniform state-space embedding vector [25–27] which is computational redundant and costs long time of computation. This drawback not only affects the accuracy in information flow detection but also limits WTE from large dataset applications.

To avoid the computational redundancy and improve the speed and accuracy, we do the extension of wavelet on two conditional mutual information measures of mixed embeddings [22, 29]. The two prototype measures are the MIME and PMIME, while the latter is the direct version of the former. These two measures are proved to be accurate and efficient in various data analysis [22, 26, 29, 35–38]. They use a progressive scheme and a stopping criterion to select only useful embedding components to be included and also to prevent false causalities [22, 29]. This stopping criterion is used as a threshold that balances the inclusion of useful lagged elements and the exclusion of useless lagged elements [22,26,29,38,46]. By using the selected non-uniform state-space embedding vectors, MIME and PMIME exclude the redundant components in information transfer evaluation, which not only guarantees the accuracy but also removes the computational redundancy.

In the simulation study, the parameters used are referenced from early studies [22, 29, 24, 38, 50]. Other parameters are plausible but depend on specific type of the applications. In this paper, we particularly studied the influence of the stopping criterion on the information transfer detection. The stopping criterion A is the proportionality of the conditional mutual information rate between the current and the past iterative cycles. We see that appropriate choices of this criterion help to ensure the true interactions to be identified, and false positiveness are prevented. Via simulation studies, we found that A = 0.97 and A = 0.95 are good choices for the stopping criterion. The stopping criterion A should not be too large or too small, because a too large A for instance A>0.97 will cause false interactions to be detected, while a too small A for instance A<0.95 will be too rigid for the criterion that it often fails to identify the interactions that truly exist. However, the choice of the stopping criterion may also depend on the datasets to be analyzed.

By definition of the WM and WPM methods, we should note that both WM and WPM evaluate the information transfer between wavelet coefficients of the same scales rather than different scales. This may be a limit of these two measures. However, the detection of information transfers across scales will be our next stage study.

In the synthetic data analysis, we note that the information transfer declines to zero for large time scales. Many reasons can explain for this phenomenon. One reason is that, when time scale grows, the frequency and resolution decrease, hence details of the time series are smeared out, the directed interaction becomes too weak to be detected. The other reason is the characteristic correlation time of theoretic maps [51–52, 54]. When the time scale exceeds the characteristic correlation time, wavelet coefficients of the coupled time series become fully correlated and the system becomes deterministic, information transfer measures such as MIME and PMIME are vanished for deterministic systems [22, 29, 50]. Another reason is that, the time scale threshold may correspond to the frequency where two time series have large mutual information between each other, the wavelet coefficients are correlated at this frequency and the system becomes deterministic. This concept of correlation is one of the key features of wavelet [24, 39–43] that makes WM and WPM special in this case. We are interested to make the bold hypothesis that the WM and WPM may be able to inference the cross-correlation time between time series and may also be able to identify the common frequencies for signal correlations.

## Conclusion

In this paper, we have proposed two multi-scale information transfer measures, namely the WM and WPM, which are the MORLET wavelet extension of conditional mutual information from mixed embedding measures. Both measures are model-free and accurate in information transfer detection of various datasets. By using non-uniform state-space embeddings, both WM and WPM are computational efficient which outperform WTE in both accuracy and speed. Due to the nature of wavelet, the proposed measures may have wide-applications including also non-stationary and discontinuous data analysis.

## Supporting information

S1 Dataset Data for the cosine map. (MAT)

S2 Dataset Data for the Henon maps. (MAT)

S3 Dataset Data for the system of three coupled variables. (MAT)

S4 Dataset Data for the Lorenz systems. (MAT)

S5 Dataset Data for the reading experiment. (MAT)

S6 Dataset Data for the fixed incomes. (MAT)
